# Compound effector genes suppress malaria parasite infections in gene-drive population modification strains of the African malaria mosquitoes, *Anopheles gambiae* and *Anopheles coluzzii*

**DOI:** 10.1093/g3journal/jkag014

**Published:** 2026-03-03

**Authors:** Rebeca Carballar-Lejarazú, Yuemei Dong, Thai Binh Pham, Taylor Tushar, Mihra Tavadia, George Dimopoulos, Anthony A James

**Affiliations:** Department of Microbiology & Molecular Genetics, University of California, Irvine, Irvine, CA 92697-4025, United States; W. Harry Feinstone Department of Molecular Microbiology and Immunology, Bloomberg School of Public Health, Malaria Research Institute, Johns Hopkins University, Baltimore, MD 21205, United States; Department of Microbiology & Molecular Genetics, University of California, Irvine, Irvine, CA 92697-4025, United States; Department of Microbiology & Molecular Genetics, University of California, Irvine, Irvine, CA 92697-4025, United States; W. Harry Feinstone Department of Molecular Microbiology and Immunology, Bloomberg School of Public Health, Malaria Research Institute, Johns Hopkins University, Baltimore, MD 21205, United States; W. Harry Feinstone Department of Molecular Microbiology and Immunology, Bloomberg School of Public Health, Malaria Research Institute, Johns Hopkins University, Baltimore, MD 21205, United States; Department of Microbiology & Molecular Genetics, University of California, Irvine, Irvine, CA 92697-4025, United States; Department of Molecular Biology & Biochemistry, University of California, Irvine, Irvine, CA 92697-3900, United States

**Keywords:** CRISPR-Cas9, antimicrobial peptides, single-chain antibodies, fitness, *Plasmodium falciparum*

## Abstract

Malaria remains a major global health burden and is caused by protozoan parasites in the genus *Plasmodium*. Parasites are transmitted to humans during blood feeding by anopheline mosquitoes, and members of the *Anopheles gambiae* species complex are important vectors in sub-Saharan Africa. Gene-drive technologies offer promising options for disease control by enabling the spread of genetic traits through mosquito populations that block parasite transmission. We report here the development and characterization of four population modification gene-drive strains in *Anopheles gambiae s.s*. and *An. coluzzii* carrying compound effector genes. We sought to enhance the effectiveness of existing gene-drive strains to block *Plasmodium* transmission, thereby reducing vector competence and minimizing the opportunities for selection of resistant parasites. Two compound effector gene modules, TP24 and TP43, were introduced using Cas9 endonuclease and dual guide RNAs into TP13-based gene-drive strains to produce the *An. gambiae* AgTP24 and AgTP43 strains. The gene-drive cassettes were then introgressed into *An. coluzzii* to produce AcTP24 and AcTP43. Gene-drive dynamics, gene conversion, and inheritance were high in all strains, with 95% to 100% inheritance of the gene-drive constructs. Life table analyses showed mixed impacts on fitness dependent on the species and copy number (hemi- or homozygosity) of the gene-drive systems. The compound effector molecule gene complexes significantly reduced both parasite prevalence and infection intensities in *An. gambiae* and *An. coluzzii* following challenge assays with the human malaria parasite, *P. falciparum*. These findings highlight the potential of compound effector strategies in gene-drive systems to achieve durable malaria transmission control.

## Graphical Abstract

**Graphical Abstract:**
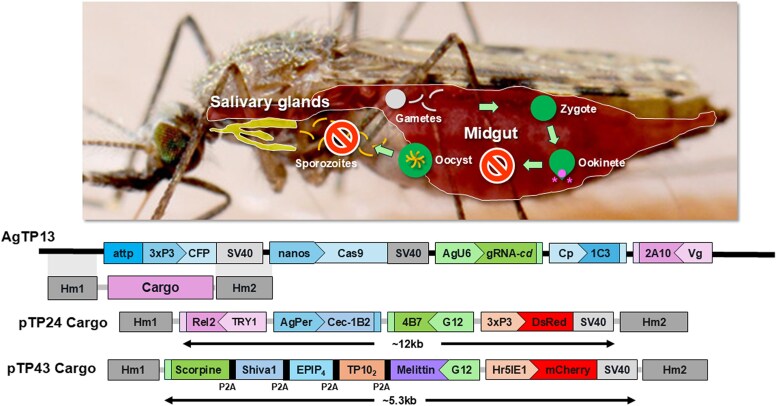

## Introduction

Novel proposed approaches to control the transmission of human malaria parasites include genetics-based strategies designed to achieve population suppression or population replacement/modification of the mosquito vectors. Population suppression tools seek to reduce the number of competent vector mosquitoes and thereby reduce the risk of human exposure to infectious bites. These methods are designed to achieve results similar to insecticide use, environmental modification to eliminate mosquito breeding sites, and personal protection, including bed nets and repellents (Nolan et al. 2011a; Nolan 2021; Xu et al. 2025). Population replacement/modification tools seek to change the ability of mosquitoes to support the development and transmission of the parasites, thereby reducing human disease and death (Carballar-Lejarazú and James 2017). Such tools require “beneficial” anti-parasite effector genes that prevent parasite development and transmission and a means to spread these genes through target vector populations quickly enough to have a meaningful epidemiological impact. The last few years have seen the development of highly-efficient CRISPR/Cas9-based gene-drive systems capable of fixing beneficial genes in target mosquito populations in laboratory cage trials within a short number of generations after their release (Gantz et al. 2015; Carballar-Lejarazú et al. 2020, 2023). While some details of the gene-drive systems still need work, it is reasonable to conclude that these could be effective when moved to the field (Eckhoff et al. 2017; James et al. 2018).

The development of efficacious anti-parasite effector genes has a long history of success because they could be tested using transgenesis technologies that predate the gene-drive systems. Indeed, some genes and their products were identified using transient expression in mosquitoes and parasite challenge assays, and this work provided support for subsequent efforts to use genetics to alter vector competence (James and Carballar-Lejarazú 2025). The anti-parasite effector genes fall into three general categories: (1) allelic variants of mosquito genes that have a negative impact on parasite development, one of the most-studied being the L224Q amino acid substitution in the fibrinogen-related protein 1 (FREP1) (Li et al. 2013; Zhang et al. 2015, 2023; Li et al. 2025), (2) induction and over-expression of genes encoding antimicrobial peptides (AMPs) and endogenous immune-system transcription regulatory factors, with genes encodings cecropins being a notable example of the former (Kokoza et al. 2000; Dong et al. 2011; Isaacs et al. 2011; Luckhart et al. 2013), and (3) introduction and expression of exogenous genes, sometime referred to as synthetic, and the engineered single-chain antibodies (scFvs) derived most often from murine monoclonal antibodies raised against parasite stage-specific surface proteins are prime examples of these (Yoshida et al. 1999; Isaacs et al. 2011, 2012; Sumitani et al. 2013).

The deployment of genetic strategies that reduce human malaria incidence (rate of new infections) and prevalence (percentage of infected individuals) requires anti-parasite effector molecules that impact parasite prevalence and intensities of infection in the mosquito vectors. Previous work identified a number of parasite reduction thresholds that, if met by any intervention strategy, are predicted to have a meaningful impact on malaria epidemiology. Modelling using an animal infection system predicted successful transmission blocking if a ≥ 32% reduction in parasite oocyst prevalence over three transmission cycles with a low infectious mosquito biting rate could be achieved (Blagborough et al. 2013). Experiments and meta-analyses of data from human and animal infection studies show a non-linear relationship between mosquito parasite burdens (intensities of infection) and subsequent vertebrate host infection, with levels of <10,000 sporozoites in mosquito salivary glands significantly decreasing infection probabilities (Aleshnick et al. 2020; Graumans et al. 2020). It is important to emphasize that these thresholds have yet to be verified for human malaria in natural transmission settings. However, while animal models are not likely to translate directly to human transmission epidemiology, they do provide initial targets for evaluating effector molecule efficacy during laboratory development stages.

One of the challenges identified for the use of modified genes in mosquitoes is the possibility that the modified/introduced gene products would confer a load that would reduce the fitness of the transgenic lines. Such loads in the gene-drive system could impact their introduction into the target vector population. A number of fitness studies have shown a range of outcomes from no or minimal effects to large ones that affected mosquito viability and reproductive traits (Catteruccia et al. 2003; Marrelli et al. 2006; Amenya et al. 2010; Bottino-Rojas et al. 2022; Dilani et al. 2022). A gene-drive system developed for *An. stephensi* created a recessive mutation in the target gene that resulted in a phenotype that severely affected survival and reproduction in homozygous females (Gantz et al. 2015; Bottino-Rojas et al. 2022). Efforts to mitigate potential fitness impacts include using the control DNA of sex-, stage-, and tissue-specific genes that confine the expression of effector gene products to “compartments” where the parasite develops in post-blood-fed female mosquitoes, and these are expected to pose no or minimal loads on males and subadult stages (Carballar-Lejarazú and James 2017). Notably, a number of transgenic mosquito strains have shown no issues when in direct competition with wild-types in controlled cage studies (Pham et al. 2019; Carballar-Lejarazú et al. 2023).

A second challenge is that the parasites will be selected for resistance to the products of the effector genes. This is a reasonable concern considering how adaptable the parasites are to selection of resistance to the many preventive and therapeutic drugs that have been developed and their long-term survival in hosts that have developed immune responses that moderate infection intensities and disease (Gatton et al. 2004; Blasco et al. 2017; Haldar et al. 2018). Approaches to prevent this have been adopted from antimicrobial drug regimens and utilize two effector genes with different target sites, thereby reducing the probability of selecting parasites resistant simultaneously to both (Carballar-Lejarazú et al. 2023).

The previously-reported autonomous Cas9/guide RNA (gRNA)-based gene-drive strains, AgTP13 in *Anopheles gambiae* and AcTP13 in *An. coluzzii*, express two anti-parasite effector gene scFvs, m1C3 and m2A10, targeting oocysts and sporozoites, respectively (Isaacs et al. 2011; Carballar-Lejarazú et al. 2023). These strains showed high drive efficiency (>95% inheritance of the gene-drive system) and significant reductions of *P. falciparum* prevalence (≥32%) and mean intensities of infection (MII) in parasite challenge assays, with the majority of the mosquitoes achieving the proposed sporozoite intensity threshold levels (<10,000 per salivary gland pair) for efficacy. Here, we modified AgTP13 and AcTP13 with additional anti-parasite effector gene cassettes to further enhance parasite refractory capacity and diminish the chance of the development of parasite resistance to the two existing scFvs. The gene cassettes, TP24 and TP43, carry genes encoding additional scFvs, AMPs, and the NF-κΒ immune system transcription factor, Rel2 (Dong et al. 2020). The strains were evaluated for gene-drive efficiency, transgene impacts on fitness, and effector molecule efficacy in parasite challenge assays. All strains had highly efficient gene-drive activity and strong and statistically significant impacts on *P. falciparum* prevalence and MIIs. However, while the added genes imposed minimal loads impacting the fitness of hemi-and homozygous *An. coluzzii* strains, they had a significant effect on the *An. gambiae* strains and prevented the establishment of homozygous colonies.

## Materials and methods

### Ethics statement

All studies were performed in compliance with the requirements of the Guide for the Care and Use of Laboratory Animals of the National Institutes of Health, and the Institutional Animal Care and Use Committee at Johns Hopkins University approved the protocol (permit no: M006H300). Mice were used only as a blood source for mosquito rearing. Commercial anonymous human blood was used for *P. falciparum* gametocyte cultures and infection assays, and informed consent was not required.

### Mosquito species, strains, and lines

Colonies of the *Anopheles gambiae* G3, a derivative strain, G3 X1 (Volohonsky et al. 2015), and the *Anopheles coluzzii* Mopti line (BEI MRA-763; BEI Resources) were used as wild-type (WT) strains and are the sources of insects in all experiments requiring wild-type crosses or comparisons with transgenic *An. gambiae* (AgTP24 and AgTP43) or transgenic *An. coluzzii* (AcTP24 and AcTP43), respectively. A colony of the AgTP13 gene-drive line was used as the docking line for the microinjection experiments (Carballar-Lejarazú et al. 2023).

*Plasmid design and construction*: All referenced oligonucleotide primer sequences are listed in Supplementary Table 1, and plasmid and inserted DNA sequences are provided in the Supplementary Material (Supplementary Table 4).

Integration of the pTP24 and pTP43 plasmids into AgTP13 lines was performed by a SWAP strategy with two sgRNAs (SWAP-1, GCGACTAGTTAATTAAAGG; SWAP-2, GCTGATTATGATCTAGAGTCG) to remove the fluorescent 3xP3-CFP region of the drive cassette of AgTP13 and insert new effector cargo along with a new red fluorescent marker gene (Adolfi et al. 2020). These gRNAs were cloned into the plasmids, pTB32 and pTP33, respectively, by annealing two pairs of oligonucleotides (TP334/TP335 and TP336/TP337) (Millipore-Sigma), followed by ligation with the Infusion Cloning kit (Takara Bio) into the pTP31 linearized with the *Bbs*I restriction endonuclease.

Plasmids pTP24 and pTP43 were cloned using the Infusion Cloning kit to assemble multiple fragments into the backbone of plasmid pTP20, which has the two AgTP13 genomic regions for homology-mediated integration of pTP24 and pTP43 into the genome location of AgTP13. pTP20 contains a left homology arm 1,067 base-pairs (bp) in length flanking exon 4 of the *cardinal* gene and a 1,138 bp right homology arm spanning the SV40 3′-end untranslated region (UTR) and part of the *An. gambiae nanos* gene promoter, with a *Pme*I restriction endonuclease cleavage site in between (Supplementary Table 4). Four fragments were cloned into pTP20 linearized with *Pme*I to generate pTP24. Fragment 1 is a Rel2 coding sequence under the *Trypsin 1* control DNA, amplified from pTP19 using primers TP292/TP288. Fragment 2, amplified with primers TP268/TP269, contains the *An. gambiae peritrophin 1* (*AgPer1*) gene control DNA, the *cecropin* gene, and an scFv, 1B2, from a Genscript-synthesized plasmid. Fragment 3, generated with primers TP270/TP271, includes the *G12* control DNA and the m4B7 scFv, also from Genscript. Fragment 4 is a 3xP3-DsRed cassette (without the SV40 region) amplified with primers TP276/TP277. Similarly, four fragments were cloned into pTP20 to produce pTP43. Fragments 1 and 2 contain the *An. gambiae G12* gene promoter, 5′- and 3′-end UTR regions amplified from *An. gambiae* genomic DNA using primer pairs TP396/TP403 and TP402/TP399, respectively. Fragment 3 is the multi-effector cassette Melittin-(TP10)_2_-(EPIP)_4_-SHIVA-Scorpine connected by a P2A linker, amplified from pEntryAgCp-MultiE-TrypT using primers TP397/TP398 (Dong et al. 2020). Fragment 4 is an Hr5IE1-mCherry cassette (without the SV40 region) amplified with primers TP394/TP395.

### Microinjection of embryos and screening procedures

Microinjections of mixtures of pTP32, pTP33, pTP24, or pTP43 were performed as described previously (Adolfi et al. 2020; Carballar-Lejarazú et al. 2021). Adult G_0_ females and males were outcrossed to WT mosquitoes of the opposite sex, and progeny screened for DsRed or mCherry fluorescence using UV-fluorescence microscopy.

### Molecular validation of gene drive cassette integration and effector molecules' integrity

Genomic integration was verified by gene amplification analysis using AgTP23 and AgTP43 genomic DNA as a template and specific primer pairs (Supplementary Table 1). For AgTP24, the right-hand junction between the right homology arm of swap integration and the cargo was amplified using primers TP280/CO53 to produce an amplicon 2,919 bp in length, and the left-hand junction between the left homology arm and the cargo was amplified using primers TP180/TP149 (2,540 bp). For AgTP43, the right-hand junction between the right homology arm of the SWAP integration and the cargo was amplified using primers TP280/CO53 to produce a fragment 4,021 bp in length, and the left-hand junction between the left homology arm and the cargo was amplified using primers TP180/TP406 (2,442 bp). Primers VgSeqF4 and TP471 were used to amplify the junction between the *cardinal* gene and the whole drive cassette, including the existing AgTP13 drive components. Primers AK5 and AK6 were used to amplify a fragment 396 bp in length of the *cardinal* gene genomic flanking region for verification of the presence of a WT allele in the hemizygous (one copy of the dominant red fluorescence gene) mosquitoes.

Genomic DNA obtained from homozygous AgTP24 and AgTP43, hemizygous AgTP24 and AgTP43, and control WT-X1 mosquitoes was extracted using the Extract-N-Amp Tissue PCR kit (Sigma-Aldrich) with modifications for mosquito samples (Pham et al. 2019). Oligonucleotide primer pairs were used in 30 µL reactions with Dreamtaq polymerase (ThermoFisher Scientific) and DNA templates from single mosquitoes. Cleaned DNA products were sent for Sanger sequencing (Genewiz) following the company's sample submission guidelines.

### Antimalarial effector gene expression profiling

Twenty-five hemizygous AgTP24 females and 25 hemizygous AgTP43 females were collected at 0 (non-fed), 4, 12, 24, and 48 h after the bloodmeal (hPBM) (5 individuals per time point) and were dissected to separate the midguts (M) and carcasses (C, all tissues remaining after removal of the midgut). Male AgTP24 and AgTP43 (5 mosquitoes each) were homogenized as a whole sample. WT-X1 females were collected at 3–4 h PBM and used as a negative control. RNA extractions were performed using adaptations of reported protocols (Isaacs et al. 2012; Gantz et al. 2015; Carballar-Lejarazú et al. 2023). Two micrograms of extracted RNA were treated in a 50 µL reaction with the DNase Treatment and Removal kit (ThermoFisher Scientific). A total of 200 ng of treated RNA was used for cDNA synthesis for each sample using the qScript cDNA synthesis kit (QuantaBio) following the manufacturer's recommended protocol. RT-PCR reactions were performed with Phusion polymerase (New England Biolabs) using the manufacturer's recommended protocol. Synthesized cDNA from AgTP24 females was amplified with gene-specific primer pairs for 1C3 (1C3-RTPCR-F, 1C3-RTPCR-R), 2A10 (2A10-RTPCR-F, 2A10-RTPCR-R), 1B2 (TB367, TP371), 4B7 (TP35, TP374), Rel2 (TP148, TP150) and RPS7 (RPS7-F, RPS7-R) to determine the expression profiles of the effector genes in the transgenic mosquitoes (Supplementary Table 1). Synthesized cDNA from AgTP43 females was amplified with gene-specific primer pairs for 1C3 (1C3-RTPCR-F, 1C3-RTPCR-R), 2A10 (2A10-RTPCR-F, 2A10-RTPCR-R), MultiE (RT-MultiE-F, RT-MultiE-R2), and RPS7 (RPS7-F, RPS7-R) to determine the expression profiles of the effector genes in the transgenics. RT-PCR experiments for hemizygous AcTP24 and AcTP43 were also carried out as described above with samples of non-fed and blood-fed females collected at 0, 4, 24, and 48 hPBM with RNA of WT-Mopti 4hPBM used as controls.

### ddPCR validation for overexpression of Rel2

ddPCR validation of Try1-Rel2 expression was done to allow comparisons of the introduced genes with the endogenous *Rel2* expression profiles. Total RNA from AgTP24 and WT-X1 female midguts and carcasses was extracted from 0 (non-fed), 4, 12, 24, and 48 hPBM samples, converted to cDNAs, and quantified using digital droplet PCR (ddPCR). The *Rel2* cDNA was amplified with primers TP364/TP365 and detected with probe Rel2-ddPCR-HEX (hexachlorofluorescein) (Supplementary Table 1) using the adapted protocol from Carballar-Lejarazú et al. (2021) with one instead of two probes. ddPCR experiments were also carried out for AcTP24 at the same time points and WT-Mopti samples for controls.

### Initial matings and gene-drive assessments

G_1_ larvae positive for the dominant red fluorescent markers in the eyes (DsRed^+^, TP24) or body (mCherry^+^, TP43) were reared to pupae and then separated individually for independent matings. Each emerged male was provided with 15 WT-X1 females, and each emerged female was provided with 10 WT-X1 males for mating in 16oz (473 mL) cups. Each G_1_ cross was allowed to mate for three days, after which the mating families were moved to small cages, and red fluorescent G_1_ female families were provided an additional 5–10 WT-X1 wild-type females to encourage blood-feeding and egg laying. Families were fed four consecutive bloodmeals and inspected daily for death of adults, and all dead adults were immediately preserved at −20 °C for molecular validation. Families that produced sufficient viable offspring for mating, had high drive inheritance rates (GDIs, described below), and were confirmed with molecular analyses to have an intact drive system, were maintained past the G_2_ generation. Male families were selected to expand and further develop both the AgTP24 and AgTP43 lines due to their high drive inheritance rates and to avoid the possibility of maternally-generated mutant alleles accumulating in the colony. The G_2_ progeny from one highly-productive male family were selected to generate the AgTP24 line, and the G_2_ progeny from two highly-productive male families were combined and used to establish the AgTP43 line. The G_2_ progeny were allowed first to intercross in small cages in order to generate homozygous AgTP24 and AgTP43 colonies. After mating for three to five days, G_2_ males were removed from the homozygous colony cages, placed in separate small cages, and provided 100 WT-X1 females to generate hemizygous colonies. The G_3_ progeny with phenotypes indicative of homozygosity, DsRed^+^/*cd^−^* or mCherry^+^/*cd^−^*, were selected and subsequently intercrossed to generate a homozygous colony. However, the homozygous colonies for both AgTP24 and AgTP43 could not able to be maintained past the fourth generation. The G_3_ progeny with phenotypes indicative of hemizygosity, DsRed^+^/*cd^+^* or mCherry^+^/*cd^+^*, were separated as pupae and kept as virgin males and females, and 50 virgin hemizygous males were subsequently mated to 100 WT-X1 females to maintain a hemizygous colony. Hemizygous colonies were maintained with this approach for all subsequent generations.

### Generation of the AcTP24 and AcTP43 lines via introgression

A total of 50 hemizygous AgTP24 (DsRed^+^/*cd^+^*) or AgTP43 (mCherry^+^/*cd^+^*) males were mated to 50 wild-type *Anopheles coluzzii* Mopti females. Progeny were recovered and screened for the hemizygous phenotype, and 50 male progeny with the hemizygous phenotype were separated as pupae, kept as virgins, and outcrossed to 50 wild-type *Anopheles coluzzii* Mopti females. This process was repeated for a total of five generations, after which one colony was maintained continuously as hemizygous according to the procedure described above, and a new colony was generated from an intercross of hemizygous males and females. After the first initial intercross of hemizygous males and females, progeny were screened and selected for a homozygous phenotype, DsRed^+^/*cd^−^* or mCherry^+^/*cd^−^*, and were kept as a continuously intercrossing colony. Every subsequent generation was screened for both hemizygous and homozygous phenotypes to verify a consistent drive inheritance in the hemizygous colony and a consistency of the homozygous phenotype in the homozygous colony.

### Male and female lineage gene-drive dynamics

Male and female lineage gene-drive dynamic experiments were performed as described (Carballar-Lejarazú et al. 2020, 2023, 2025). Briefly, hemizygous AgTP24, AcTP24, AgTP43, and AcTP43 males and females recovered from a continuously outcrossing colony of transgenic males to wild-type females were used as the parental mosquitoes for the first set of crosses. The hemizygous AgTP24, AcTP24, AgTP43, and AcTP43 males and females were outcrossed to wild-type mosquitoes of the opposite sex and corresponding wild-type strain, WT-X1 or Mopti. The progeny of these outcrosses were screened for eye phenotypes and the presence/absence of the red fluorescent markers at the pupal stage. Gene-drive system inheritance (GDI) is measured as the percentage of red fluorescent-positive progeny of the total in any cross in which one or both parents carry one or two copies of the gene-drive system (Carballar-Lejarazú et al. 2025). Hemizygous gene-drive system mosquitoes outcrosses to wild-types were used to calculate the percentage of progeny inheriting an HDR-mediated “converted” gene drive target allele (cGDI) as the percentage of progeny carrying the gene-drive system in excess of what would result from Mendelian segregation (GDI-50)/50X100), and is an index of gene drive efficiency (also referred to as “homing”; Hammond et al. 2016).

Male and female pupae with a phenotype indicative of hemizygosity, DsRed^+^/*cd^+^* or mCherry^+^/*cd^+^*, were separated, kept as virgins, and subsequently outcrossed to wild-types of the opposite sex and respective wild-type strain. Male and female gene drive lineages were observed for two generations to determine drive efficiency, stability, and the generation of any exceptional or unexpected eye phenotypes.

### Life-table parameters

Life-table parameters were evaluated as described for the AgTP13, AcTP13, and AcNosCd-1 lines in Carballar-Lejarazú et al. (2023). Experiments to determine each parameter were performed in triplicate. Hemizygous AgTP24 and hemizygous AgTP43 were compared to the WT-X1 strain; the cumulative data acquired over a total of three replicates for AgTP24 and AgTP43 and six replicates for WT-X1 were used in the statistical analyses. Hemizygous and homozygous AcTP24 and AcTP43 were compared to the wild-type Mopti strain. Experiments to determine life-table parameters for hemizygous AcTP24 and hemizygous AcTP43 were run in parallel with wild-type Mopti, and experiments to determine life-table parameters for homozygous AcTP24 and homozygous AcTP43 were run in parallel with wild-type Mopti at a separate time point. The cumulative data acquired over all replicates for each line were used in the statistical analyses. Statistical analyses for each life-table parameter were performed using R (version 4.2.2), and the same statistical tests were used for each parameter as described in Carballar-Lejarazú et al. (2023).

### *Plasmodium falciparum* infection assays and statistical analysis

Adult female mosquitoes from the following genotypes were used: hemi-AgTP24, hemi-AgTP43, and their respective controls (WT-G3 or WT-X1), as well as hemi- and homozygous AcTP24, AcTP43, and the WT-Mopti control line. All mosquitoes were maintained under standard insectary conditions at 27 ± 1 °C, 70 to 80% relative humidity, with a 12:12 h light:dark photoperiod. Females were sugar-fed until the infectious bloodmeal. The NF54 strain of *P. falciparum* was cultured in human O⁺ erythrocytes under standard conditions until mature stage V gametocytes were obtained. Cultures were diluted to achieve target gametocytemia levels for experimental challenges and designated “Low” (0.01–0.03% gametocytemia, representative of many natural transmission levels) and “High” (0.15–0.3% gametocytemia, used to increase statistical power in laboratory assays). Infectious bloodmeals were prepared by combining cultured gametocytes with fresh human serum and erythrocytes. Bloodmeals were maintained at 37 °C and offered to mosquitoes for 60 min using a membrane-feeding apparatus fitted with parafilm following previously established protocols (Tripathi et al. 2020). Fully engorged females were separated within 1 h post-feeding and maintained on 10% sucrose for the duration of the parasite development period.

Oocyst counts were obtained from midguts dissected at 7–8 d post-infection, stained with 0.1% mercurochrome, and examined under a light microscope at 100× or 200× magnification. The number of oocysts per midgut was recorded for each mosquito. Sporozoite counts were obtained from salivary glands dissected 14–16 d post-infection. Glands were homogenized in phosphate-buffered saline (PBS), and numbers were determined using a hemocytometer and light microscopy.

Dot plots of parasite counts (oocysts and sporozoites) were generated in GraphPad Prism version 10. MII and median values were calculated for each group. Prevalence (percentage of infected mosquitoes) and intensity data from independent biological replicates were pooled prior to analysis to maintain balanced sample sizes across treatments. Infection intensity was compared between experimental and control groups using the Mann–Whitney *U* test. Infection prevalence was compared using Fisher's exact test. Differences were considered statistically significant at *P* < 0.05. Data and statistical outputs are presented in Supplementary Tables 2 and 3.

## Results

### Design, construction, and generation of the AgTP24 and AgTP43 gene-drive lines

The design features of the TP24 and TP43 constructs are based on previous work that utilized the control DNA, promoters and 5′- and 3-end UTRs, from genes whose products are expressed in the mosquito midguts to target either the gametocyte or oocyst stages of the malaria parasites, and those that are expressed in the fat body and whose products accumulate in the mosquito hemolymph and target the sporozoite stage parasites (Bottino-Rojas and James 2022). Effector genes were chosen based on previous studies that showed their ability to reduce parasite prevalence and MII (Dong et al. 2011, 2020; Isaacs et al. 2011; Carballar-Lejarazú et al. 2023). All scFvs used have known targets that are either parasite enzymes or surface proteins important for parasite development and invasion. Their general mode of action is to prevent the parasites from developing from one stage to another (*e.g.* gametocyte to oocyst; oocyst to ookinete) and/or invading mosquito tissues (midgut and salivary glands). The products of the immune-regulatory and AMP-encoding genes are known to lyse parasites (Dong et al. 2020).

**Fig. 1.**
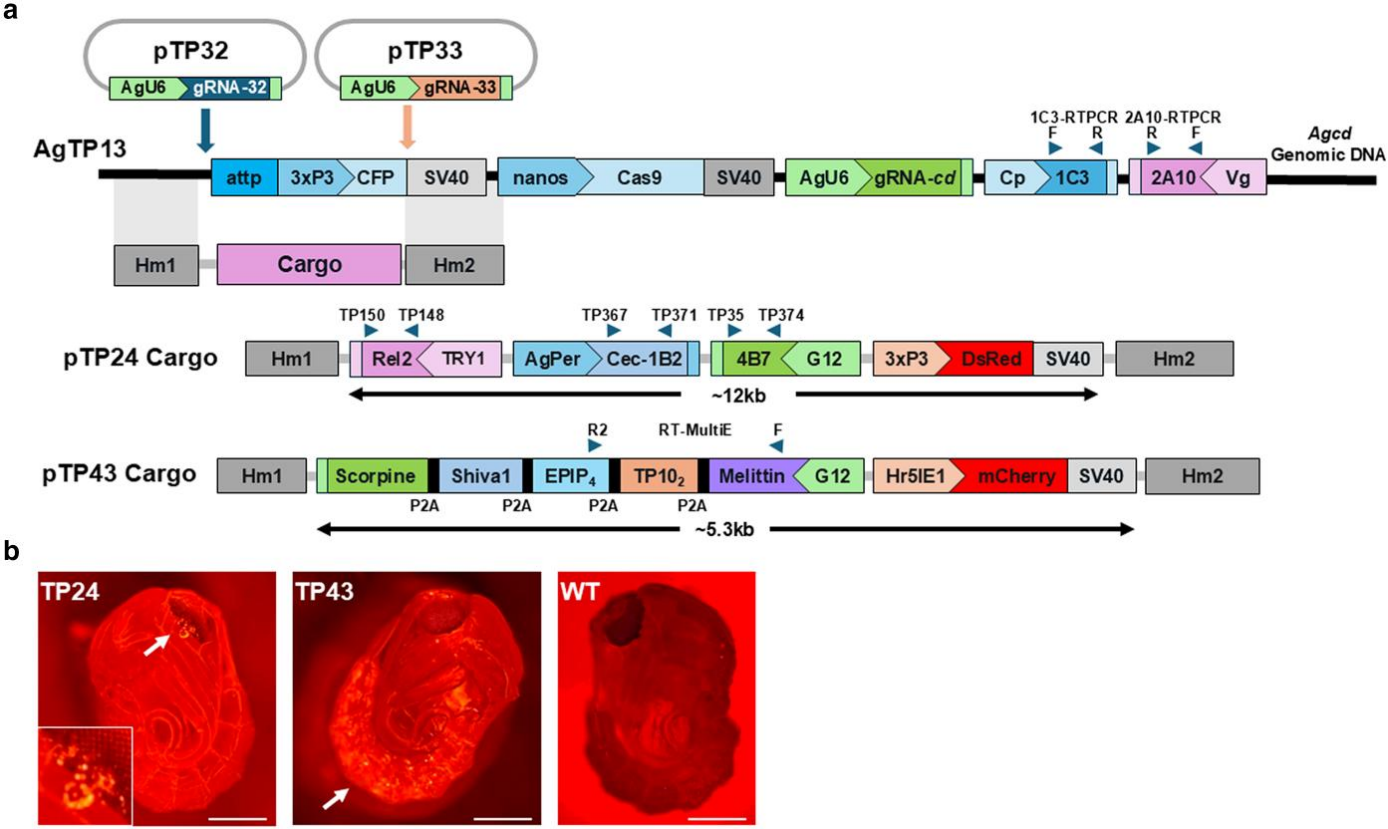
AgTP24 and AgTP43 swap strategy. Cas9/gRNA-mediated cassette exchange: a) Two gRNAs encoded in the plasmids pTP32 and pTP33 guided the cleavage (down-facing arrows) in the AgTP13 genome, resulting in the excision of a 1,141 bp fragment including the 3xP3-CFP marker gene. The plasmids pTP24 and pTP43 carrying homology arms (gray boxes, Hm1, Hm2) flanking both cut sites were used as templates for homology-directed repair (HDR) to promote the insertion of the DsRed (TP24) or mCherry (TP43) marker genes and two new sets of effector genes. pTP24 cargo components include the left- and right-homology arms (gray boxes, Hm1, Hm2), the dominant DsRed fluorescent protein eye marker cassette (lighter-red boxes, 3xP3, DsRed) and SV40 3′-end DNA (light gray box), the *G12* gene control DNA (promoter and 5′- and 3′-end UTR sequences) driving the m4B7 scFv (green boxes, G12, 4B7), the *An. gambiae Peritrophin 1* gene control DNA driving the expression of Cecropin and 1B2 (light-blue boxes, AgPer, Cer-1B2) and the *An. Gambiae Trypsin 1* gene control DNA driving the expression of Rel2 (light-pink boxes, Try1, Rel2). The pTP43 cargo components include the left- and right-homology arms (gray boxes, Hm1, Hm2), the dominant mCherry fluorescent protein body marker gene (lighter-red boxes, Hr5EI1, mCherry), and SV40 3′-end DNA, the *G12* gene control DNA driving the expression of the multi-effector cassette (MultiEff; Dong et al. 2020) comprising Mellitin-(TP10)2-(EPIP)4-SHIVA-Scorpine connected by P2A linkers (black boxes). The names, relative location and orientation of oligonucleotide primers used in transcription assays (RTPCR) are shown above each sequence (arrowheads). b) Images of AgTP24, AgTP43 and control wild-type pupae under red-fluorescent imaging light microscopy. The phenotypes are subtle with the TP24 fluorescence seen in the eye (white arrow and inset) and the TP43 fluorescence distributed throughout the body and seen best in the abdomen (white arrow). Scale bar = 1 mm. Key and abbreviations: 1C3, 2A10, 4B7, DNA encoding scFvs; 3xP3, triplicate copy of *P3* gene promoter and 5′-end enhancer sequences; AgPer, *An. gambiae Peritropin-1* midgut-specific gene control DNA; AgU6, *An. gambaie* U6 gene encoding sequence; attP, phage *P1* attachment site; Cas9, CRISPR-associated protein 9; cd, *cardinal* gene gRNA sequence; Cec, *Cecropin A* encoding sequence; CFP, cyan fluorescent protein; Cp, *An. gambiae carboxypeptidase A* midgut-specific gene control DNA; DsRed, *Discosoma* sp. red fluorescent protein-encoding gene; EPIP, *enolase-plasminogen-interaction-peptide* encoding sequences; F, “forward” oligonucleotide primer; G12, *An. gambiae G12* midgut-specific gene control DNA; gRNA, guide RNA encoding sequence; Hm, homology arm; MultiE, multieffector; nanos, *An. gambiae nanos* gene control DNA; P2A, self-cleaving proteolytic protein-encoding sequence; R, “reverse” oligonucleotide primer; Rel2, *Relish 2* transcription factor; SV40, simian virus 40 3′-end untranslated region; TP10, transportan 10 encoding sequence; Try1, *Trypsin 1* midgut-specific gene control DNA; Vg, *An. stephensi Vitellogenin 1* fat body-specific gene control DNA.

The *An. gambiae* gene-drive strains, AgTP24 and AgTP43, were generated by inserting DNA cassettes encoding multiple anti-parasite effector genes into AgTP13 with a SWAP approach that involves the coordinated action of two gRNAs transcribed from the pTP32 and pTP33 plasmids in the presence of a donor template (pTP24 or pTP43) that carries a dominant fluorescent marker gene, the effector-gene coding sequences, and DNA homology arms matching the flanking regions of each of the cut sites (Fig. 1a; Adolfi et al. 2020). This approach also results in the excision of the AgTP13 3xP3-CFP eye marker gene.

The pTP24 donor template is ∼12 kilobase (kb) pairs in length and contains four linked components: (1) a gene encoding an scFv, 1B2, targeting parasite gametocytes, fused with an AMP-encoding gene, *Cecropin A* (*CecA*), and driven by the *An. gambiae Peritropin-1* (*AgPer1*) midgut-specific gene control DNA, (2) the m4B7 scFv gene targeting oocysts under the control of the *G12* midgut-specific gene control DNA, (3) the mosquito immune gene, *Relish 2* (*Rel2*) driven by the *Trypsin 1* (*Try1*) midgut-specific gene control DNA, and (4) a *Discosoma* sp. red fluorescent protein encoding gene (DsRed) expressed by the 3xP3 promoter (Fig. 1b; Matz et al. 1999; Horn and Wimmer 2000; Dong et al. 2011; Isaacs et al. 2011). The *AgPer1* control DNA sequences confer constitutive transcript expression in adult females, whereas the control DNAs of the *G12* and *Try1* genes regulate transcript expression in females at different times after a bloodmeal (Abraham et al. 2005; Nolan et al. 2011b).

The pTP43 donor template is ∼5.3 kb in length and consists of two components: (1) a multiple effector cassette (MultiEff) containing the coding sequences of four AMPs (melittin, shiva1, transportan 10 [TP10], and scorpine, each separated by a self-cleaving proteolytic protein sequence, P2A), and the enolase-plasminogen-interaction-peptide (EPIP) coding sequence, all under the control of the *G12* gene control DNA, and (2) the commonly-used Hr5IE-mCherry gene cassette as a dominant fluorescent body marker (Dong et al. 2020). TP10 and EPIP are present as tandem copies of two and four DNA coding sequences, respectively.

#### AgTP24 line

Microinjection of 1,630 AgTP13 embryos with a mixture of the pTP24, pTP32, and pTP33 plasmids resulted in 405 (24.8%) surviving G_0_ adults (Table 1). These were outcrossed to the *An. gambiae* X1 strain, designated here as WT-X1, resulting in 16 DsRed-positive (DsRed^+^) transgenic G_1_ larvae (0.07%, 16/22,783) from a G_0_ male family and 13 of these, six males and seven females, survived to adulthood. Only four males and five females produced viable DsRed^+^ progeny, and these were used as described below to establish the AgTP24 line (Fig. 1b). The two G_1_ males that did not produce progeny were found to have an incorrect orientation of the pTP24 insertion.

**Table 1.** Generation and establishment of the AgTP24 and AgTP43 compound effector strains.

	No. G_0_ embryos injected	No. G_0_ embryos surviving to adults	No. G_0_ adults outcrossed	No. G_1_ progeny scored	No. G_1_ red fluorescent progeny^a^	No. G_1_ larvae survive to adults
**AgTP24**	1,630	405	405	22,783	16 (0.07%)	13 (6♂, 7♀)
**AgTP43**	5,086	211	211	9,548	39 (0.41%)	11 (4♂, 8♀)^b^
**Rec*kh*** ^c^	504	259	184	25,293	96 (0.38%)	2 (1♂, 1♀)

^a^Red fluorescence, TP24: DsRed^+^ or TP43: mCherry^+^.

^b^Pooled results from two G_0_ families.

^c^Data from Adolfi et al. (2020).

The fertile G_1_ DsRed^+^ males and females were mated individually to 15 or 10, respectively, WT-X1 members of the opposite sex for three days, after which five WT-X1 females were added to the transgenic female cages to encourage blood feeding and egg laying. Following initial characterizations of the gene-drive efficiencies described below, DsRed^+^ G_2_ mosquitoes from the male family that produced the most progeny were used to establish the AgTP24 lines, as this was anticipated to decrease the probability that mutant target alleles produced by maternal effects could impact drive system dynamics (Carballar-Lejarazú et al. 2022, 2025). All DsRed^+^ G_2_ progeny were allowed first to intercross to establish a homozygous colony, and then the DsRed^+^ G_2_ males were removed and outcrossed again to WT-X1 females to establish a hemizygous colony. However, a considerable genetic load affecting G_3_ female fertility and fecundity was observed in the intercrosses, and only a hemizygous line could be maintained thereafter by outcrossing.

Initial assessments of the AgTP24 gene-drive efficiency could be derived from the crosses used to establish the line. Efficiency values include the gene-drive drive inheritance, GDI, measured as the percentage of DsRed^+^ progeny of the total, and the gene-drive conversion inheritance, cGDI, which is the percentage of progeny carrying the gene-drive system in excess of what would be expected by Mendelian segregation of the alternative gene-drive and wild-type alleles (Materials and methods; Carballar-Lejarazú et al. 2025). All four G_1_ AgTP24 males that produced viable offspring had high GDIs, with 100% of the G_2_ progeny inheriting a copy of the drive system and all target alleles converted (cGDI = 100%; Table 2). The GDIs and cGDIs of the five G_1_ AgTP24 fertile females could not be determined because, following mating of them to wild-type males, additional WT-X1 females were added to each cage to promote feeding and egg laying.

**Table 2.** Summary of initial drive-system inheritance in AgTP24 and AcTP43 larval phenotypes of G_2_ outcrosses to wild-type mosquitoes.

Line	Founder	G_2_ adult phenotypes^a,b^		
		red^+^/cd^+^	red*^−^*/cd^+^	Total^c^
**AgTP24**	**G_1_ 1** ♂	88	0	88 (100%)
	**G_1_ 2** ♂	135	0	135 (100%)
	**G_1_ 3** ♂	18	0	18 (100%)
	**G_1_ 4** ♂	68	0	68 (100%)
	**G_1_ 1**♀	38	65	103
	**G_1_ 2**♀	80	102	182
	**G_1_ 3**♀	118	80	198
	**G_1_ 4**♀	54	33	87
	**G_1_5**♀	158	116	274
**AgTP43**	**G_1_ 1**♂	181	0	181 (100%)
	**G_1_ 2**♂	170	0	170 (100%)
	**G_1_ 1**♀	11	215	226
	**G_1_ 2**♀	1	255	256
	**G_1_ 3**♀	17	235	252
	**G_1_ 4**♀	12	179	191
	**G_1_5**♀	24	24	48

^a^Progeny of single G_1_ adults outcrossed to wild-types of the opposite sex.

^b^G_3_ progeny positive for red fluorescence (TP24: DsRed^+^ or TP43: mCherry).

^c^Female crosses were supplemented by the addition of wild-type females to promote mating and egg deposition.

#### AgTP43 line

Similar protocols for generating the AgTP24 strain were used to establish the AgTP43 strain. The 211 (4.14%) surviving G_0_ adults following injection of 5,086 embryos with the pTP43, pTP32, and pTP33 plasmids were outcrossed to WT-X1 mosquitoes of the opposite sex (Table 1). A total of 39 mCherry^+^ transgenic larvae (0.41%, 39/9,548) were recovered from two different G_0_ female families, 35 from one and four from another (Fig. 1b). Eight G_1_ larvae, four males and four females, survived to adulthood from one female family, and three G_1_ female larvae survived to adulthood from the other female family. Two each G_1_ adult males and females from the larger family, and all three G_1_ females from the smaller family, produced viable mCherry^+^ progeny. The outcrossed hemizygous G_1_ male matings had high GDIs and cGDIs (100% for each, values for females could not be determined due to the addition of WT-X1 females), and the lines were maintained successfully in subsequent generations (Table 2). The mCherry^+^ G_2_ offspring from the two males that produced offspring were combined to establish the AgTP43 line.

Similar to the AgTP24 protocol, mCherry^+^ G_2_ progeny were mated in inter- and outcrosses to produce homozygous and hemizygous AgTP43 colonies, respectively. However, as with the AgTP24 efforts, multiple attempts to generate a homozygous AgTP43 colony through intercrossing hemizygous individuals beyond the G_3_ failed as homozygous larval and pupal mortality prevented recovery of adult mosquitoes.

### Molecular validation of AgTP24 and AgTP43 genome integration

**Fig. 2.**
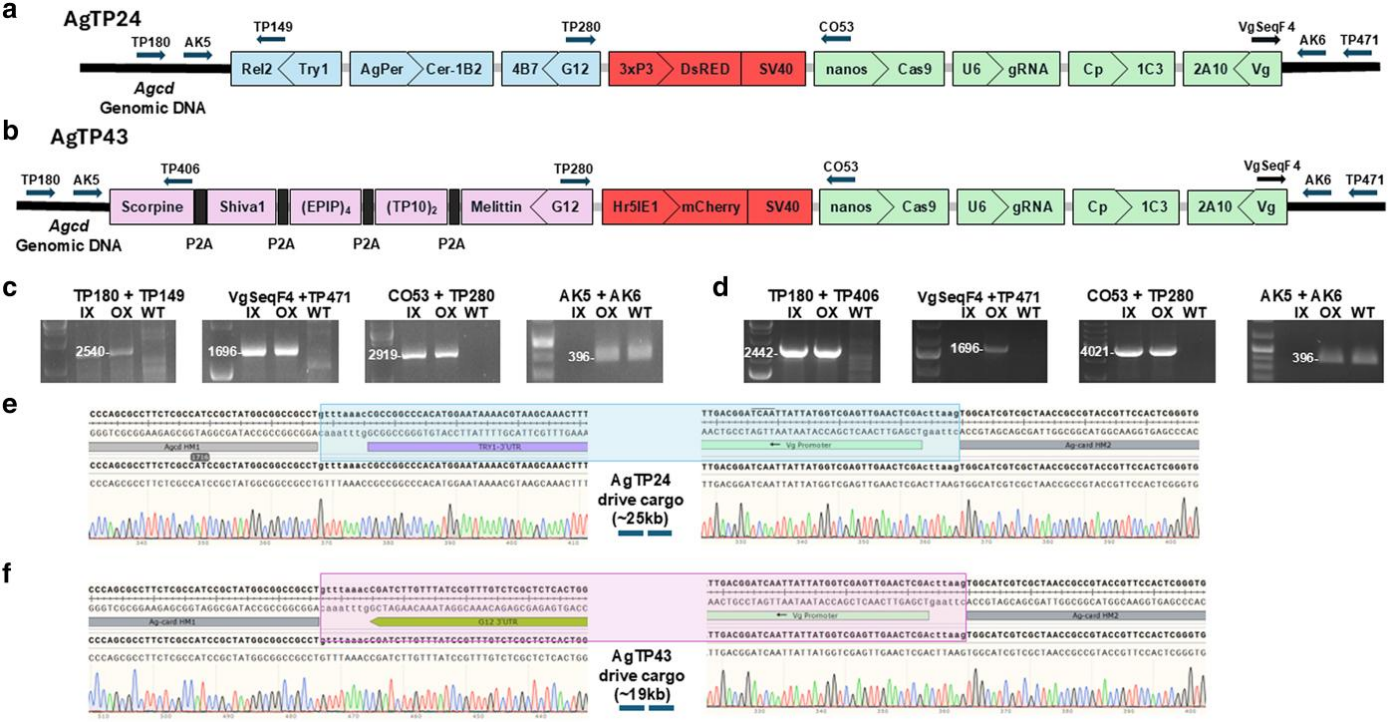
Molecular validation of AgTP24 and AgTP43. Schematic representation of AgTP24 (a) and AgTP43 (b) at the *cardinal* locus (not to scale). Gene amplification analysis of the integrated AgTP24 (c) and AgTP43 (d) gene-drive system in *An. gambiae*. Oligonucleotide primer names (Supplementary Table 1), orientations with respect to the constructs, and approximate locations are indicated with blue arrows (above). Gel electrophoresis and staining images (left to right) of amplicons between the left junction and cargo, right junction and cargo, right junction of swap integration into AgTP13, and left and right junctions of the flanking region in the *cardinal* locus. Oligonucleotide primer pairs are listed above each image, and the source of the genomic DNA template is indicated (outcross [OX, hemizygous], intercross [IX, homozygous], and WT-X1 [WT] control DNA). Expected amplicon sizes in base pairs (bp) verified by DNA sequencing are listed on the left side of each gel image. Sequencing results showing the accurate integration of the cargo of AgTP24 (e) and AgTP43 (f) at the intended location in the third exon of the *An. gambiae cardinal* gene (*Agcd*). Boxed and labeled regions correspond to representations in the schematic above. Abbreviations are listed in Fig. 1.

The pTP24 and pTP43 insertions in the AgTP13 line were confirmed by sequencing amplicons generated from genomic DNA from transgenic progeny of both outcrossed (OX) and intercrossed (IX) AgTP24 and AgTP43 individuals using junction-specific oligonucleotide primers (Fig. 2; Supplementary Table 1). The results verified Cas9/gRNA-mediated homology-directed repair (HDR) at the two designed gRNA cleavage sites that resulted in precise integration and orientation of the pTP24 and pTP43 gene cassettes. The resulting gene-drive cargoes, which now include the newly-introduced components from SWAP integration along with the existing AgTP13 elements (AgNos-Cas9, U6gRNA, Cp-m1C3, and Vg-m2A10), are ∼25 kb (AgTP24) and ∼19 kb (AgTP43) in length.

### Generation and characterization of the AcTP24 and AcTP43 gene drive lines

The AgTP24 and AgTP43 gene-drive systems were introgressed into the wild-type *An. coluzzii* Mopti (WT-Mopti) strain to evaluate effector gene and drive system performance in a sibling species of the *An. gambiae* species complex. Introgression was achieved by crossing 50 AgTP24 or AgTP43 hemizygous males to 50 WT-Mopti females. Male offspring from the outcross with a red fluorescent (DsRed^+^ or mCherry^+^), black-eye (*cd^+^*) phenotype were separated as pupae and kept for 3–5 d as virgins after adult emergence, after which they were mated with another 50 WT-Mopti females. This process was repeated five times to yield a population carrying an estimated ∼98% of the *An. coluzzii* genome excluding the Y chromosome. The actual introgression percentage is likely higher because transgene drive is high despite known suppression of meiotic recombination due to inversion polymorphisms (Pescod et al. 2023; Tushar et al. 2024). Following the outcrosses, one colony each of the TP24 or TP43 gene-drive mosquitoes was maintained continually as hemizygous lines by outcrossing males to WT-Mopti females.

Homozygous AcTP24 and AcTP43 lines were established using progeny with only red fluorescent (DsRed^+^ or mCherry^+^), red-eye (*cd^−^*) phenotypes selected after an initial hemizygous intercross, and these were allowed to interbreed until all subsequent generations consisted of only DsRed^+^/*cd^−^* or mCherry^+^/*cd^−^* progeny, thereby verifying that the colony contains mosquitoes with two copies of the respective gene drive system in each individual. Unlike AgTP24 and AgTP43, AcTP24 and AcTP43 were both maintained successfully as homozygous colonies.

### Life-table parameters and fitness assessments

Hemizygous and homozygous transgenic lines were compared with their respective control lines for a number of life-table parameters that could affect their fitness.

#### AgTP24 fitness assessments

Larval and pupal stage AgTP24 hemizygotes develop slightly faster than their WT-X1 counterparts with a total of 7.2 d required on average to complete all larval development stages (1st to 4th instars, L1-L4) and an average time to pupal eclosion of 8.2 d compared to the respective control values of 7.4 and 8.5 d (Table 3). Despite the moderately accelerated development times, there were no significant differences in larval viability (AgTP24, 78%; WT-X1, 77%) and the sex ratios of emerged adults. However, despite robust immature stages, reductions were observed in AgTP24 adult fitness parameters. Male and female hemizygous AgTP24 mosquitoes had reduced respective longevity, 25 d and 27 d, compared to corresponding WT-X1 controls, 32 and 30 d. Furthermore, hemizygous AgTP24 females had lower fecundity, 68 eggs/batch, and fertility, a 67% hatch rate, compared to WT-X1 (95 eggs/batch and a 78% hatch rate). AgTP24 males also were slightly less successful reproductively in one-to-one competition with WT-X1 males, contributing 47.4% of the offspring compared to the 52.6% produced by WT-X1 males, and this value serves as a proxy for gene drive-carrying males competing successfully with control males for access to females.

**Table 3.** Comparisons of life parameters of wild-type, hemizygous AgTP24 and AgTP43, and hemizygous and homozygous AcTP24 and AcTP43 mosquitoes.^a^.

Species	*Anopheles gambiae*			*Anopheles coluzzii*				
Line	Wild-type WT-X1	AgTP24 (HEMI)	AgTP43 (HEMI)	Wild-type Mopti	AcTP24 (HEMI)	AcTP24 (HOM)	AcTP43 (HEMI)	AcTP43 (HOM)
**Larval development in days ± SD**	7.4 ± 0.75	7.2 ± 0.85*	6.6 ± 0.64***	8.0 ± 1.2	7.4 ± 0.8***	9.0 ± 1.4***	8.2 ± 1.0	7.1 ± 0.8***
**Pupal development time in days ± SD**	8.5 ± 0.74	8.2 ± 0.87**	8.1 ± 0.66***	9.2 ± 1.1	8.9 ± 0.9	10.4 ± 1.6***	9.0 ± 1.0	8.3 ± 0.8***
**Larval viability ± SD**	0.77 ± 0.04	0.78 ± 0.05	0.87 ± 0.03*	0.74 ± 0.10	0.87 ± 0.02*	0.69 ± 0.07	0.76 ± 0.07	0.79 ± 0.02
**Percent female adults ± SD**	49.6% ± 7.0%	49.1% ± 4.4%	47.9% ± 1.2%	51.8% ± 1.2%	48.7% ± 2.6%	48.6% ± 5.2%	50.0% ± 3.5%	48.1% ± 4.1%
**Percent male adults ± SD**	50.4% ± 7.0%	50.9% ± 4.4%	52.1% ± 1.2%	48.2% ± 1.2%	51.3% ± 2.6%	51.4% ± 5.2%	50.0% ± 3.5%	51.9% ± 4.1%
**Adult female longevity in days (95% CI)**	32 (30, 33)	27** (26, 29)	33 (31, 35)	22 (21, 23)	21 (19, 24)	17**** (15, 19)	18*** (15, 19)	18*** (17, 21)
**Adult male longevity in days (95% CI)**	30 (27, 32)	25** (23, 26)	30 (24, 33)	23 (22, 25)	18**** (16, 19)	14**** (12, 16)	25 (24, 27)	27* (25, 30)
**Fecundity ± SD**	95 ± 36	68 ± 26***	87 ± 35	93 ± 36	75 ± 26*	94 ± 41	97 ± 37	85 ± 28
**Fertility (Eggs hatched) ± SD**	78% ± 21%	67% ± 30%*	80% ± 18%	69% ± 28%	62% ± 30%	64% ± 28%	76% ± 29%	82% ± 27%***
**IX Male contribution ± SD (number of mosquitoes/total)**	—	—	—	(vs AcTP24) 50.9% ± 8.6% (1,631/3,257) (vs AcTP43) 43.8% ± 9.9% (2,359/5,418)	—	49.1% ± 8.6% (1,626/3,257)	—	56.2% ± 9.9%*** (3,059/5,418)
**OX Male contribution ± SD (number of mosquitoes/total)**	(vs AgTP24) 52.6% ± 3.7% (2,895/5,500) (vs AgTP43) 52.6% ± 7.6% (1,740/3,311)	47.4% ± 3.7%** (2,605/5,500)	47.4%± 7.6%* (1,571/3,311)	(vs AcTP24) 49.1% ± 2.1% (1,278/2,590) (vs AcTP43) 22.9% ± 3.1% (776/3,369)	50.9% ± 2.1% (1,312/2,590)	—	77.1% ± 3.1%**** (2,593/3,369)	—

^a^All statistical analyses are performed as comparisons of respective wild-types with the gene-drive lines. All experiments are triplicate biological replicates.

*P-value = 0.05–0.001; **P-value = 0.001–0.0001; ***P-value = 0.0001–0.00001; ****P-value < 0.00001. HEMI: hemizygotes, HOM: homozygotes.

#### AcTP24 fitness assessments

AcTP24 larval and pupal development differed between the homo- and hemizygous strains (Table 3). Hemizygous AcTP24 mosquitoes were similar to AgTP24 hemizygotes in having slightly faster larval (7.4 vs 8.0 d) and pupal (8.9 vs 9.2 d) development times compared to the WT-Mopti. In comparison, AcTP24 homozygotes took slightly longer to develop through the larval (9.0 vs 8.0 d) and pupal (10.4 vs 9.2 d) stages compared to controls. Hemizygous AcTP24 had improved larval viability with 87% surviving through all developmental stages compared to 74% WT-Mopti, but there was no difference between AcTP24 homozygotes and controls. No differences in the sex ratios of emerged adults were observed between any of the AcTP24 lines and WT-Mopti.

AcTP24 adults displayed reduced longevity with a more pronounced effect in homozygotes, with males and females having shorter median lifespans of 14 and 17 d, respectively, compared to corresponding WT-Mopti controls with 23 and 22 days (Table 3). In contrast, a reduced lifespan, 18 d, was observed only for AcTP24 hemizygote males and not females (21 d), but the reduction is not as pronounced as that seen with AcTP24 homozygote males. AcTP24 adult male and female reproductive parameters were better than those observed for AgTP24. Hemizygous AcTP24 females had reduced fecundity, 75 eggs/batch, when compared to WT-Mopti females, 93 eggs/batch, no other significant differences in fertility or fecundity were observed between the WT-Mopti and the AcTP24 lines. AcTP24 males were as likely to contribute to the next generation as WT-Mopti males.

#### AgTP43 fitness assessments

Few fitness impacts were observed in the hemizygous AgTP43 line despite the inability to form a thriving homozygous colony (Table 3). Similar to AgTP24 hemizygotes, AgTP43 hemizygotes developed more quickly during the immature stages, 6.6 d for larval development and 8.1 d for pupae to eclose, compared to 7.4 d for larvae and 8.5 d for pupae in the WT-X1 controls. AgTP43 hemizygotes showed higher larval viability with 87% of the larvae surviving through all development stages compared to 77% for the WT-X1 controls. There were no significant differences in fitness in most of the measured adult parameters, sex ratio, longevity, fertility, and fecundity when comparing AgTP43 and WT-X1. However, similar to AgTP24, AgTP43 males were slightly less likely to contribute to the next generation, with only 47.4% of offspring being contributed by AgTP43 hemizygous males compared with the 52.6% contributed by WT-X1 wild-type males in one-to-one competition.

#### AcTP43 fitness assessments

AcTP43 fitness assessments differed from those seen with AgTP43. Homozygous AcTP43 had shorter larval, 7.1 d, and pupal, 8.3 d, development times compared to WT-Mopti, 8.0 d and 9.2 d, respectively (Table 3). However, there were no significant differences from controls in the immature development times of hemizygous AcTP43 mosquitoes. Differences in larval viability were not observed for either hemizygous or homozygous AcTP43 when compared to WT-Mopti controls. Adult-stage fitness parameters were much more varied in AcTP43 compared to WT-Mopti than what was observed for AgTP43 and WT-X1. Female hemizygous and homozygous AcTP43 had reduced lifespans, medians of 18 d for both lines, when compared to the median 22 d of WT-Mopti. Male hemizygous and homozygous AcTP43 had longer lifespans, medians of 25 and 27 d, respectively, compared to a median of 23 d for WT-Mopti males. AcTP43 females showed no statistically significant differences in fecundity compared to WT-Mopti females but had better fertility with hatch rates of 76% (hemizygous) and 82% (homozygous) compared to the 69% hatch rate of WT-Mopti females. AcTP43 males had substantially improved reproductive fitness, with hemizygous and homozygous AcTP43 males contributing 77.1 and 56.2% of the offspring in the next generation when in one-to-one competition with WT-Mopti males.

### Gene-drive properties

#### Male and female lineage AgTP24 and AcTP24 gene-drive dynamics

AgTP24 and AcTP24 male and female lineages were assessed as described previously for gene-drive efficiency and emergence of target gene mutations (Carballar-Lejarazú et al. 2020, 2023, 2025). Briefly, hemizygous AgTP24 and AcTP24 males and females were outcrossed for two generations (F1 and F2) to WT-X1 and WT-Mopti mosquitoes, respectively, of the opposite sex (Table 4). Progeny phenotypes from the hemizygous outcrosses were recorded to measure drive inheritance (presence/absence of red fluorescence marker; GDI), HDR-mediated converted target alleles (cGDI), and eye colors (tear and *cardinal* red eyes) associated with target allele mutations. “Tear” phenotypes are eyes that are phenotypically mosaic for the wild-type, *cardinal* red-eye, and fluorescent marker phenotypes. “Expected” phenotypes are those whose frequencies and/or presentation are predicted by canonical Mendelian segregation and inheritance, whereas “exceptional” refers to those that are not (*e.g.,* tear/mosaic phenotypes).

**Table 4.** Gene drive inheritance and conversion efficiencies and eye phenotypes from male- and female-derived AgTP24 and AcTP24 lineages.

Strain	AgTP24				AcTP24			
Gen	Male-founder lineage drive GDI (cGDI) efficiency^a,b^		Female-founder lineage drive GDI (cGDI) efficiency^a,b^		Male-founder lineage drive GDI (cGDI) efficiency^a,b^		Female-founder lineage drive GDI (cGDI) efficiency^a,b^	
**F1**	♂ 99.4% (1,175/1,182) (98.8%)		♀ 94.9% (1,051/1,107) (89.8%)		♂ 98.5% (1,147/1,164) (97%)		♀ 98.6% (1,243/1,261) (97.2%)	
**F2**	♂ 99.8% (1,119/1,121) (99.6%)	♀ 91.5% (367/401) (83%)	♂ 100% (837/837) (100%)	♀ 87.4% (174/199) (74.8%)	♂ 99.6% (776/779) (99.2%)	♀ 99.9% (961/962) (99.8%)	♂ 97.8% (1,229/1,257) (95.6%)	♀ 97.5% (627/643) (95%)

Gen	Male-founder lineage *cardinal* eye^c^		Female-founder lineage *cardinal* eye^c^		Male-founder lineage *cardinal* eye^c^		Female-founder lineage *cardinal* eye^c^	
**F1**	♂ 0% (0/1,182)		♀ 0.4% (4/1,107)		♂ 0% (0/1,164)		♀ 0.95% (12/1,261)	
**F2**	♂ 0% (0/1,121)	♀ 0.7% (3/401)	♂ 0% (0/837)	♀ 0.7% (1/199)	♂ 0% (0/779)	♀ 0.3% (3/962)	♂ 0% (0/1,257)	♀ 0% (0/643)

Gen	Male lineage tear-eyed^d^		Female lineage tear-eyed^d^		Male lineage tear-eyed^d^		Female lineage tear-eyed^d^	
**F1**	♂ 0% (4/1,182)		♀ 2.6% (29/1,107)		♂ 0.3% (4/1,164)		♀ 12.7% (160/1,261)	
**F2**	♂ 0% (0/1,121)	♀ 5.8% (23/401)	♂ 0% (0/837)	♀ 5.0% (10/199)	♂ 0.4% (3/779)	♀ 7.7% (74/962)	♂ 0.6% (7/1,257)	♀ 9.2% (59/643)

^a^Symbols refer to the sex of the gene-drive parents: ♂ male gene-drive parents; ♀ female gene-drive parents.

^b^Drive inheritance efficiency (GDI) was determined by calculating the percentage of progeny expressing the DsRed marker gene out of the total originating from the mating event (numbers below); drive conversion efficiency (cGDI) is the percentage of progeny carrying the gene-drive system in excess of what would result from Mendelian segregation.

^c^Eye phenotype was determined by calculating the percentage of heteroallelic progeny containing one copy of the drive element (DsRed^+^) and one resistant mutant *cardinal* (*cd^−^*) allele out of the total originating from the mating event.

^d^Eye phenotype was determined by calculating the percentage of progeny showing the tear phenotype out of the total originating from the mating event.

Hemizygous F1 and F2 AgTP24 males had higher GDI rates, 99.4% to 100% than the corresponding hemizygous females, 87.4% to 94.9% (χ^2^ = 23.1, *P*-value < 0.0001) and higher cGDI rates, 98.8% to 100% vs 74.8% to 89.8%, respectively, both trends observed previously for the AgNosCd-1- and AgTP13-based gene drive lines (Table 4; Carballar-Lejarazú et al. 2020, 2023, 2025). In contrast, the AcTP24 GDI rates did not vary between F1 and F2 hemizygous males (97.8% to 99.6%) and females (97.5% to 99.9%; χ^2^ < 0.0001, *P*-value > 0.999), and these also are consistent with what was seen previously with AcTP13 (Carballar-Lejarazú et al. 2023). The cGDI rates reflected these inheritance values, ranging from 95.6% to 99.2% in males and 95.0% to 99.8% in females.

Both parental and F1 AgTP24 and AcTP24 hemizygous females generated progeny with exceptional phenotypes during outcrosses that are indicative of maternal-effect, drive system-generated mutant target alleles (Table 4). The range, 0.3% to 0.95%, in percentages of F1 and F2 *cardinal* red-eye phenotypes overlapped those in the progeny of female AgTP24 hemizygotes (0.4–0.7%) and AcTP24 hemizygotes (0.3–0.95%, [χ^2^ < 0.0001, *P*-value > 0.999]). The mosaic tear eye phenotype was slightly more prevalent in the progeny of hemizygous AcTP24 females, 7.7% to 12.7%, than hemizygous AgTP24 females, 2.6% to 5.8% (χ^2^ = 5.8, *P*-value = 0.02). Overall, the proportions of offspring with mosaic tear eye and *cardinal* red-eye phenotypes from hemizygous TP24 female lineages are within the range of what was observed previously for the *An. gambiae* and *An. coluzzii* NosCd-1 and TP13 lines (Carballar-Lejarazú et al. 2020, 2023, 2025).

#### Male and female lineage AgTP43 and AcTP43 gene-drive dynamics

Male and female lineages of the AgTP43 and AcTP43 lines were assessed for drive efficiency and emergence of drive-system-mediated mutations as described for the TP24 lines (Table 5). Consistent with observations from other AgNosCd-1-based gene*-*drive lines, F1 and F2 AgTP43 hemizygous males had higher GDIs, 99.0% to 100%, than hemizygous AgTP43 females, 86.2–95.0% (χ^2^ = 21.3, *P*-value < 0.0001; Carballar-Lejarazú et al. 2020, 2023, 2025). Hemizygous F1 and F2 AgTP43 cGDIs were also consistent with previous results with males ranging from 98.0% to 100% and females from 72.4% to 90.0%. AcTP43 F1 and F2 male GDIs ranged from 99.8% to 100% and females from 97.2% to 99.2% (χ^2^ = 3.1, *P*-value = 0.08). The F1 and F2 AcTP43 cGDIs ranged from 99.6% to 100% in males and 94.4% to 96.6% in females.

**Table 5.** Gene drive inheritance and conversion efficiencies and eye phenotypes from male- and female-derived AgTP43 and AcTP43 lineages.

Strain	AgTP43				AcTP43			
Gen	Male-founder lineage drive GDI (cGDI) efficiency^a,b^		Female-founder lineage drive GDI (cGDI) efficiency^a,b^		Male-founder lineage drive GDI (cGDI) efficiency^a,b^		Female-founder lineage drive GDI (cGDI) efficiency^a,b^	
**F1**	♂ 99.0% (1,090/1,101) (98%)		♀ 95.0% (1,248/1,313) (90%)		♂ 100% (1,229/1,229) (100%)		♀ 98.3% (1,115/1,134) (96.6%)	
**F2**	♂ 100% (802/802) (100%)	♀ 93.3% (812/853) (86.6%)	♂ 99.8% (859/861) (99.6%)	♀ 86.2% (661/767) (72.4%)	♂ 100% (670/670) (100%)	♀ 99.2% (519/523) (98.4%)	♂ 99.8% (804/806) (99.6%)	♀ 97.2% (410/422) (94.4%)

Gen	Male-founder lineage *cardinal* eye^c^		Female-founder lineage *cardinal* eye^c^		Male-founder lineage *cardinal* eye^c^		Female-founder lineage *cardinal* eye^c^	
**F1**	♂ 0% (0/1,101)		♀ 0.2% (3/1,313)		♂ 0% (0/1,229)		♀ 0% (0/1,134)	
**F2**	♂ 0% (0/802)	♀ 0.4% (3/853)	♂ 0% (0/861)	♀ 0% (0/767)	♂ 0% (0/670)	♀ 0% (0/523)	♂ 0% (0/806)	♀ 0% (9/422)

Gen	Male lineage tear-eyed^d^		Female lineage tear-eyed^d^		Male lineage tear-eyed^d^		Female lineage tear-eyed^d^	
**F1**	♂ 0.6% (7/1,101)		♀ 8.9% (117/1,313)		♂ 0% (0/1,229)		♀ 4.5% (51/1,134)	
**F2**	♂ 0.5% (4/802)	♀ 6.2% (53/853)	♂ 0% (0/861)	♀ 5.1% (39/767)	♂ 0.9% (6/670)	♀ 3.4% (18/523)	♂ 0.6% (5/806)	♀ 2.1% (9/422)

^a^Symbols refer to the sex of the gene-drive parents: ♂ male gene-drive parents; ♀ female gene-drive parents.

^b^Drive inheritance efficiency (GDI) was determined by calculating the percentage of progeny expressing the DsRed marker gene out of the total originating from the mating event (numbers below); drive conversion efficiency (cGDI) is the percentage of progeny carrying the gene-drive system in excess of what would result from Mendelian segregation.

^c^Eye phenotype was determined by calculating the percentage of heteroallelic progeny containing one copy of the drive element (mCherry^+^) and one resistant mutant *cardinal* (*cd^−^*) allele out of the total originating from the mating event.

^d^Eye phenotype was determined by calculating the percentage of progeny showing the tear phenotype out of the total originating from the mating event.

There was no statistically significant difference in the proportion of mosaic tear eye phenotypes generated among AgTP43 male and female lineage hemizygous females, 5.1% to 8.9%, compared to AcTP43 hemizygous females, 2.1% to 4.5% (χ^2^ = 3.0, *P*-value = 0.09). Interestingly, no *cardinal* red-eye phenotypes were detected in the progeny from hemizygous AcTP43 female outcrosses, and the low rates, 0% to 0.4%, from the AgTP43 hemizygous females were not significantly different from the AcTP43 hemizygous females (χ^2^ < 0.0001, *P*-value > 0.999).

### Effector gene expression

**Fig. 3.**
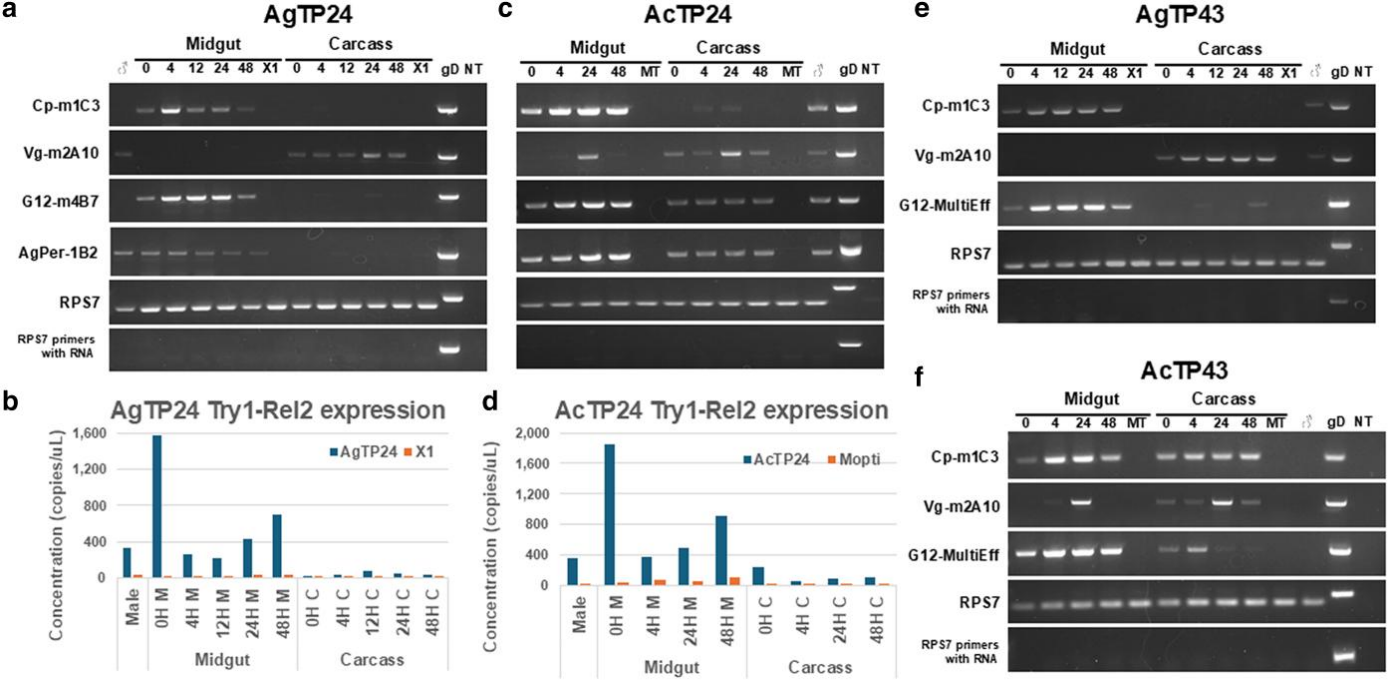
Relative effector gene transcript abundance in hemizygous AgTP24, AgTP43, AcTP24, and AcTP43 females before and after a bloodmeal. Total RNAs isolated from the midguts and carcasses of pre-bloodfed (0) and post-bloodfed females from hemizygous AgTP24 (a), AcTP24 (c), AgTP43 (e), and AcTP43 (f) at 4, 12, 24 and 48 hPBM, were used as templates for gene amplification (RT-PCR) reactions using oligonucleotide primer pairs shown in Fig. 1 and listed in Supplementary Table 1, and the results visualized in 1% agarose gels. ddPCR protocols were used to compare the relative abundance of transgene-expressed *Rel2* gene transcripts from the AgTP24 (b) and AcTP43 (d) samples at the same time points with those produced from the endogenous gene in controls (X1, WT-X1; Mopti, WT-Mopti). Abbreviations: AgPer, *An. gambiae Peritropin-1* midgut-specific gene control DNA; Cp, *An. gambiae carboxypeptidase A* midgut-specific gene control DNA; G12, *An. gambiae G12* midgut-specific gene control DNA; Try1, *Trypsin 1* midgut-specific gene control DNA; Vg, *An. stephensi Vitellogenin 1* fat body-specific gene control DNA; m1C3, m4B7, m2A10 and 1B2, scFv amplified fragments; MultiEff, multi-effector amplified fragment; RPS7, ribosomal protein 7 control amplified fragment; gD, genomic DNA; H, hours post-bloodmeal; NT, non-template control.

The expression and transcript abundance profiles of the newly-introduced effector genes, m4B7 and 1B2, in AgTP24, the multi-AMP-EPIP effector (MultiEff) cassette in AgTP43 and the existing m1C3 and m2A10 scFvs from the AgTP13 background, were determined by transcript amplification (RT-PCR) at 0 (non-fed) and 4, 12, 24, and 48 h post bloodmeal (hPBM) using total RNA isolated from midguts and carcasses (all tissues except midguts) from hemizygous AgTP24 and AgTP43 females (Fig. 3). Transcript expression and abundance assessments of the Try-Rel2 gene construct are confounded by the presence and activity of the endogenous *Rel2* gene. Therefore, digital droplet PCR (ddPCR) was used to detect any transgene-mediated enhanced transcript accumulation by comparing it with values obtained from non-transgenic (Mopti) controls.

The m1C3 and 2A10 transcript accumulation profiles in AgTP24 were similar to those observed previously in AgTP13, with m1C3 having its highest abundance in midguts at 4 hPBM and 2A10 being most abundant in the carcass samples, which include the fat body tissues, at 24 hPBM (Fig. 3a; Carballar-Lejarazú et al. 2023). Similar to m1C3, the m4B7 transcript was observed in non-fed female midgut samples but increased after a bloodmeal, with its highest abundance at 24 hPBM. 1B2 expression mediated by the *AgPer1* control DNA was midgut-specific and constitutive (seen at all time points). Rel2 transcript accumulation levels were highest in the non-fed samples, but levels were higher compared to WT-X1 controls at all time points (Fig. 3b). Based on amplicon presence and stain intensities in the gels, AcTP24 showed qualitatively greater constitutive and ectopic (non-targeted) accumulation of the effector gene transcripts than AgTP24, particularly for the AgPer1-1B2 construct, which is no longer restricted to the midgut (Fig. 3c, d).

The bloodmeal-induced transcript accumulation of m1C3 and m2A10 in AgTP43 was not regulated as precisely as AgTP24, however, the enhanced abundance based on amplicon stain intensity of the two effector transcripts at 4–48 hPBM in midgut and carcass samples potentially increased the amount of the gene product available to interact with its parasite targets (Fig. 3e). The compound effector cassette had a similar transcript abundance profile as m4B7 in AgTP24 because they both are driven by the *G12* gene control DNA with enhanced transcript accumulation seen in 4, 12 and 24 hPBM midgut samples. While amplicon staining intensity showed qualitatively greater AcTP43 constitutive and ectopic accumulation of the effector gene transcripts than AgTP43, the results verified that the antimalarial gene transcripts are present in both strains (Fig. 3e, f).

### AgTP24, AgTP43, and AcTP24 and AcTP43 effector-gene impacts on *P. falciparum*

Similar to the AgTP13 transgenic mosquitoes, the multiple effector molecule complexes in AgTP24 and AgTP43 constructs are designed to be regulated transcriptionally by the control DNA derived from genes that are expressed specifically in adult females, and with the exception of the constitutive AgPer1/1B2 module, are activated following a bloodmeal (Carballar-Lejarazú et al. 2023). This regulation is intended to ensure spatiotemporal expression patterns that optimize their activity against different *Plasmodium* developmental stages while potentially reducing the burden of impact of transgene expression on males and non-blood-fed female stages. Furthermore, previous work has shown that providing additional bloodmeals following the infectious one helps sustain effector gene transcription during successive parasite developmental stages (Carballar-Lejarazú et al. 2023). Three supplementary, uninfected bloodmeals were provided at 5, 9, and 11 d after the initial infectious feed to maintain high transcript levels from the transgene cassettes (Carballar-Lejarazú et al. 2023). This approach is supported by studies that show that some anopheline species, including *An. gambiae* and *An. coluzzii* take multiple bloodmeals in each gonotrophic cycle as part of their reproductive behavior (Briegel and Hörler 1993; Koella et al. 1998). In laboratory conditions, ∼70% of *An. gambiae* females take a second bloodmeal within 24 h of the first despite being fully engorged, and all females have taken an additional meal by day 6 (Nirmala et al. 2005). Furthermore, *Plasmodium*-infected mosquitoes are more likely than uninfected ones to take multiple bloodmeals, thus reinforcing the rationale for the experimental protocol used here (Koella et al. 1998).

Hemizygous AgTP24 and AgTP43, and both hemi- and homozygous AcTP24 and AcTP43 adult females were fed either low- or high-density gametocyte cultures (0.01–0.03% and 0.15–0.3% gametocytemia, respectively, Materials and methods). The lower parasite dose generated oocyst MIIs similar to naturally-observed field levels, while the higher dose was selected to increase statistical power in laboratory assays (Rosenberg 2008; Dong et al. 2009, 2020; Bousema et al. 2012; Isaacs et al. 2012; Gonçalves et al. 2017; Bradley et al. 2018; Soumare et al. 2021). Homozygous AgTP24 and AgTP43 lines could not be tested due to the previously-noted elevated mortality in sub-adult stages and reduced fertility and fecundity.

**Fig. 4.**
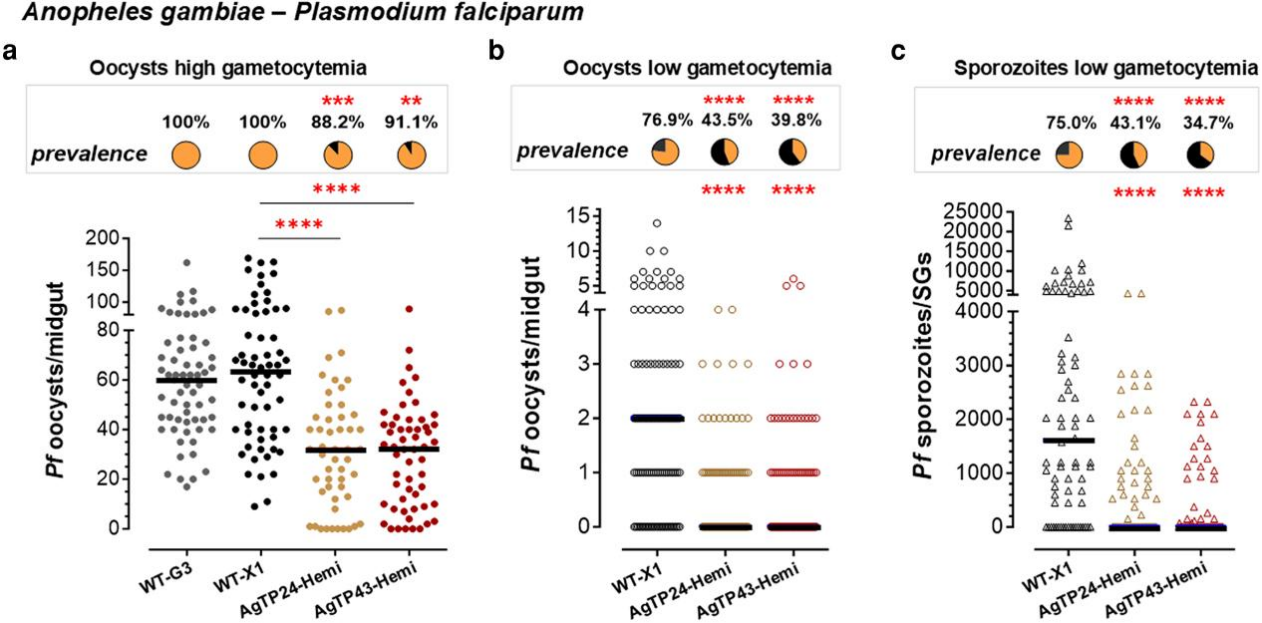
Parasite prevalence and infection intensities in hemizygous AgTP24 and AgTP43 bloodfed females following challenges with *Plasmodium falciparum* (NF54). a) Prevalence and infection intensities of oocysts in hemizygous AgTP24, AgTP43, and control WT-G3 and WT-X1 bloodfed females at high (0.15–0.30%) gametocytemia levels. b) Frequency distribution, prevalence, and infection intensities of oocyst numbers in hemizygous AgTP24, AgTP43, and control WT-G3 and WT-X1 bloodfed females at low (0.01–0.03%) gametocytemia levels. c) Prevalence and infection intensities of sporozoites in hemizygous AgTP24, AgTP43, and control WT-G3 and WT-X1 bloodfed females at low (0.01–0.03%) gametocytemia levels. Dot-plots and pie-charts display oocyst (a, b) and sporozoite (c) infection intensities and prevalence at 8 d post infection (dpi) in the midgut or 14 d dpi in the salivary glands, respectively. The horizontal black lines indicate the median values. The Fisher's Exact test was used to calculate *P*-values for infection prevalence, and the Mann–Whitney test was used for mean intensities of infections; *P < 0.05; **P < 0.01, ***P < 0.001, ****P < 0.0001. Abbreviations: Ctl, control; Hemi, hemizygous; Pf, *Plasmodium falciparum*; SGs, salivary glands.

Three biological replicates were carried out for AgTP24 and AgTP43 using hemizygous females and the control WT-X1 line with both low- and high-gametocytemia feeds. Data from these replicates were pooled (Fig. 4a: high gametocytemia; Fig. 4b, c: low gametocytemia). Under high gametocytemia conditions, oocyst prevalence dropped from 100% in controls to 88.2% (AgTP24) and 91.1% (AgTP43) (Fig. 4a, *P* < 0.001 and *P* < 0.01, respectively; full statistical summaries are provided in Supplementary Table 2). Under low infection conditions, prevalence was reduced from 76.9% in controls to 43.5% (AgTP24) and 39.8% (AgTP43), corresponding to ∼43.4% and ∼48.3% reductions, respectively (Fig. 3b, *P* < 0.0001 for both). At low infection, oocyst MIIs fell from 2.2 in controls to 0.7 in both AgTP24 and AgTP43 hemi-females, with median values decreasing from 2.0 in controls to zero for both lines (Fig. 4b, *P* < 0.0001 for both).

AgTP24 and AgTP43 hemizygous females also showed highly significant reductions in salivary gland sporozoite prevalence and infection intensity following low gametocytemia challenges (Fig. 4c). Prevalence declined from 75.0% in controls to 43.1% (AgTP24; ∼42.6% reduction) and 34.7% (AgTP43; ∼53.7% reduction) (Fig. 3c, *P* < 0.0001 for both lines; Supplementary Table 2). The sporozoite MIIs dropped from 3,112.5 in controls to 664.6 (AgTP24) and 381.8 (AgTP43), with medians reduced from 1,612.5 in controls to zero in both (Fig. 3c, *P* < 0.0001). None of the experimental groups exhibited any high sporozoite loads (≥10,000).

**Fig. 5.**
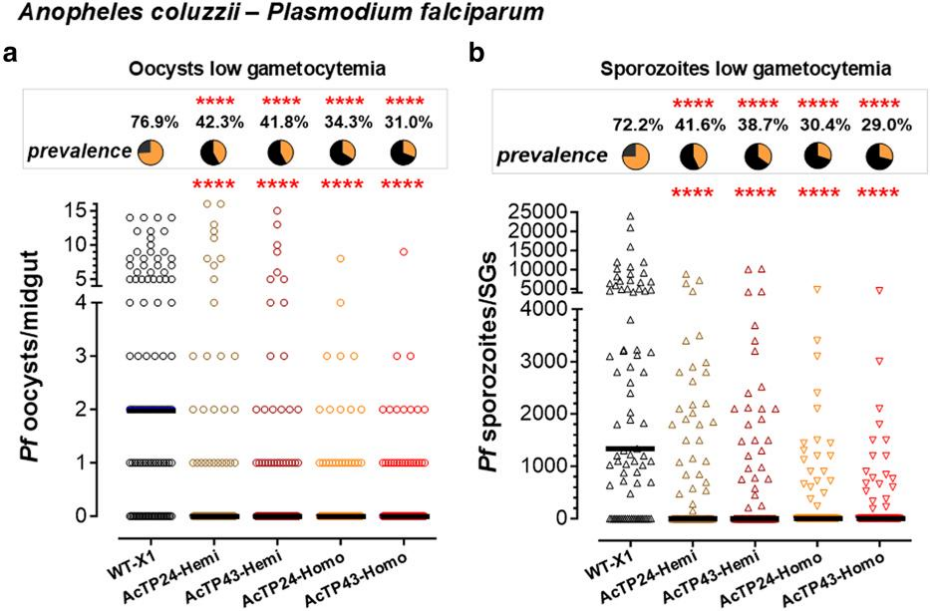
Parasite prevalence and infection intensities in hemi- and homozygous AcTP24 and AcTP43 bloodfed females following challenges with *Plasmodium falciparum* (NF54). a) Prevalence and infection intensities of oocysts in hemizygous and homozygous AcTP24, AcTP43, and control WT-X1 bloodfed females at low (0.01–0.03%) gametocytemia levels. b) Prevalence and infection intensities of sporozoites in hemizygous and homozygous AcTP24, AcTP43, and control WT-X1 bloodfed females at low (0.01–0.03%) gametocytemia levels. Dot-plots and pie-charts display oocyst (a) and sporozoite (b) infection intensities and prevalence at 8 d post infection (dpi) in the midgut or 14 d dpi in the salivary glands, respectively. The black horizontal lines indicate the median values. The Fisher's Exact test was used to calculate *P*-values for infection prevalence, and the Mann–Whitney test was used for mean intensities of infections; *P < 0.05; **P < 0.01, ***P < 0.001, ****P < 0.0001. Abbreviations: Ctl, control; Hemi, hemizygous; Homo, homozygous; Pf, *Plasmodium falciparum*; SGs, salivary glands.

As described previously, hemi- and homozygous AcTP24 and AcTP43 females have robust feeding and survival, and this allows challenges of both with *P. falciparum*-infected blood. Low gametocytemia challenges were performed to mimic natural field conditions. Three biological replicates for each genotype (hemi-, homozygous, and WT-Mopti control mosquitoes) were performed separately. When pooled, the data showed significant reductions in the prevalence of both oocyst- and sporozoite-infected mosquitoes (Supplementary Table 3). Oocyst infection prevalence decreased from 73.8% in controls to 42.3% (AcTP24 hemizygous; ∼42.8% reduction) and 41.8% (AcTP43 hemizygous; ∼43.4% reduction) (Fig. 5a). Homozygotes exhibited even stronger reductions, 34.3% (AcTP24; ∼53.6% reduction) and 31.0% (AcTP43; ∼58.1% reduction), with median intensities reduced from 2 in controls to zero in all experimental groups (*P* < 0.0001). Oocyst MIIs dropped from 4.0 in controls to 1.9 (AcTP24 hemi) and 1.5 (AcTP43 hemi), and to 0.6 and 0.5 in the respective homozygous lines.

Control sporozoite infection prevalence (72.2%) was reduced to 41.6% (AcTP24 hemi; ∼42.4% reduction) and 38.7% (AcTP43 hemi; ∼46.4% reduction), and to 30.4% (AcTP24 homo; ∼57.8% reduction) and 29.0% (AcTP43 homo; ∼59.8% reduction) (Fig. 5b, *P* < 0.0001; Supplementary Table 3). MIIs were reduced from 3,378 sporozoites per salivary gland pair in controls (WT-Mopti) to 968 (AcTP24 hemi), 918 (AcTP43 hemi), 443 (AcTP24 homo), and 342 (AcTP43 homo). Median sporozoite counts dropped from ∼1,335 in controls to zero in all experimental lines (Fig. 5b, *P* < 0.0001). Consistent with the reductions in oocysts, homozygous AcTP24 and AcTP43 lines exhibited the strongest *P. falciparum* sporozoite suppression, reducing prevalence from 72.2% in controls to ∼30% (∼58–60% reduction). While a small proportion of hemi- and homozygous mosquitoes carried relatively high parasite loads (up to ∼4,800 and ∼4,500, respectively), none (0/69) exceeded 10,000 sporozoites.

Comparisons of the impact on parasite infection prevalence and MIIs of the compound effector genes in the TP24- and TP43-containing strains with the results of previous work with the dual scFv effector genes, m1C3 and m2A10, in *An gambiae* and *An. coluzzii*, and the MultiEff AMP cassette in *An. stephensi* are complicated by potential stochastic effects (*e.g.,* gametocytemia variability, strain-specific transgene expression) that differ among the independent studies (Dong et al. 2020; Carballar-Lejarazú et al. 2023). As described, all strains carrying antiparasite effector genes reduced the oocyst and sporozoite infection prevalence in all mosquito strains, with the greatest effects seen in those carrying two copies, homozygous, of the gene cassette (Table 6). Remarkably, both hemi- and homozygous transgenic lines in all studies reduced median oocyst numbers to zero in low (0.01–0.03%) gametocytemia challenges. Furthermore, with the exception of the AcTP13/AcTP43 hemizygous comparison, all TP24- and TP43-containing strains showed greater reductions in the oocyst MIIs compared to their respective TP13 and MultiEff counterparts. It is important to note that oocyst and sporozoite counts are not distributed normally and often contain outliers (such as highly-infected midguts or salivary glands) that can drive the mean to a higher value, and this likely accounts for the AcTP13/AcTP43 hemizygous result (Dong et al. 2020). The data in aggregate support the conclusion that there is an enhanced effect on oocysts of having the TP24 and TP43 effector molecules added to the TP13-containing lines.

**Table 6.** Comparisons of dual and compound effector antiparasite gene cassettes on *Plasmodium falciparum* prevalence and mean intensities of infection.

	AgTP13^a,b^			AcTP13^a,b^			AgTP24^a^		AgTP43^a^		AcTP24^a^		AcTP43^a^		MultiEff^a,c^	
	Ctl^d^	Hemi	Homo	Ctl^e^	Hemi	Homo	Ctl^d^	Hemi	Hemi	Ctl^e^	Hemi	Homo	Hemi	Homo	Ctl^f^	Homo
Oo: pre (%)	74.2	44.2	33.9	75.2	40.0	20.3	76.9	43.6	39.8	76.9	42.3	34.3	41.8	31.0	83	45
Oo median	**2**	**0**	**0**	**2**	**0**	**0**	**2**	**0**	**0**	**2**	**0**	**0**	**0**	**0**	**3.5**	**0**
Oo: MII	4.4	2.0	1.2	2.3	0.6	0.3	2.2	0.7	0.7	4	1.9	0.6	1.5	0.6	4.8	2.2
% red Oo MII**^g^**	—	**54.5**	**72**.**7**	—	**73**.**9**	**86**.**9**	—	**68**.**1**	**68**.**1**	—	**52**.**5**	**85**.**0**	**62**.**5**	**85**.**0**	—	**54.1**
Sp: pre (%)	73.5	42.3	30.9	75.4	26.2	18.5	75.0	43.1	34.7	72.2	41.6	38.7	30.4	29.0	75	46
Sp median	**3,592**	**1,832**	**759**.**6**	**2,722**	**303**.**2**	**182**.**9**	**1,612**	**0**	**0**	**1,335**	**0**	**0**	**0**	**0**	**2,300**	**0**
Sp: MII	4,007	1,950	1,800	2,635	303.2	182.9	3,112	664.6	318.8	3,379	968.1	443.6	918.3	342.9	3,448.8	2,363.9
% red Sp MII**^g^**	—	**51.3**	**55**.**0**	—	**88**.**5**	**93**.**0**	—	**78**.**6**	**89**.**7**	—	**71**.**3**	**86**.**8**	**72**.**8**	**89**.**8**	—	**31.4**

Abbreviations: Ctl, control; Hemi, hemizygous; Homo, homozygous MII, mean intensities of infection; *n*, number of mosquitoes; Oo, oocysts; pre, prevalence; Sp, sporozoites.

^a^Low, 0.01–0.03, gametocytemia infection challenge.

^b^Data are from Carballar-Lejarazú et al. (2023).

^c^Data are from Dong et al. (2020).

^d^Controls are the *Anopheles gambiae* AgNosCd-1 strain.

^e^Controls are the *Anopheles coluzzii* WT-Mopti strain.

^f^Controls are an *Anopheles stephensi* wild-type strain.

^g^Percent reductions (red) in oocyst (Oo) and sporozoite (Spo) mean intensities of infection (MII).

Not surprisingly, the additive effects of the compound genes on sporozoite numbers reflect what is seen with oocysts. Sporozoite prevalences are greatly reduced in all strains challenged with the low gametocytemia bloodmeal and meet threshold objectives (Table 6). Most notably, the medians are reduced to zero in all TP24- and TP43-containing lines, showing an impact of the transgene effector additions in TP24 and TP43-containing lines that surpasses that of the TP13 lines. Concomitant reductions in sporozoite MIIs are seen. Here again, the data support the conclusion that there is an additive effect on sporozoites of having the TP24 and TP43 effector molecules combined with those of the TP13 complement.

## Discussion

Four novel population modification lines, AgTP24, AcTP24, AgTP43, and AcTP43, were generated in two members of the *Anopheles gambiae* species complex, an *An. gambiae* X1 line derived from the G3 laboratory strain that was colonized from The Gambia and is an *An. gambiae ss/An. coluzzii* hybrid, and Mopti, a long-colonized *An. coluzzii* strain originating in Mali (Volohonsky et al. 2015; Anopheles gambiae 1000 Genomes Consortium 2020). The SWAP procedures used to generate the AgTP24 and AgTP43 lines are not as efficient (measured as the percentage of insertions per embryo injected) as primary integration procedures using either transposable elements or Cas9/gRNA-mediated HDR, but they were effective in integrating large DNA fragments and expanding the size of the exogenously-derived DNA at the insertion site, and the AgTP43 SWAP compares favorably with previous work in *An. stephensi* (Adolfi et al. 2020; Table 1).

Genetic barriers exist that impact the introgression of genes from one member of the *An. gambiae* species complex into another, and these are due in large part to chromosomal inversion polymorphisms that interfere with homologous pairing during meiosis and suppress recombination (Sharakhov and Sharakhova 2024; Sharakhova and Sharakhov 2025). However, gene-drive systems have been shown to be remarkably resilient to this barrier. In one study, efficient drive was seen in outcrosses between a number of *An. gambiae* strains with chromosomal inversion polymorphisms and high levels of genome variation near the gRNA target site (Pescod et al. 2023). Another study looked at the introduction and introgression of the AgNosCd-1 gene-drive system from which the core TP13, TP24, and TP43 drive elements are derived into different genetic backgrounds (Tushar et al. 2024). The overall drive inheritance in fertile F1 hybrid intercrosses in three geographically diverse *An. gambiae* strains and one *An. coluzzii* colony was 100% in males and ∼99% in females, far exceeding canonical Mendelian inheritance ratios. These combined data show the robustness of some gene-drive systems in the presence of chromosomal polymorphisms in sibling species.

The highly efficient drive-system inheritance rates (GDI) seen in the hemizygous AgTP24, AcTP24, AgTP43, and AcTP43 male and female lineages are similar to those observed for the AgNosCd-1, AcNosCd-1, AgTP13, and AcTP13 drive strains (Carballar-Lejarazú et al. 2020, 2023, 2025). Both paternal and maternal effects resulting from deposition of Cas9/gRNA complexes into early embryos impact drive inheritance by creating drive-resistant mutant target alleles (Carballar-Lejarazú et al. 2025). This is evident in the calculation of the target allele conversion rate, cGDI, which is usually lower than the GDI. A higher rate of generation of drive-resistant mutant alleles in female lineages contributes to the observed differences in male/female drive dynamics. However, the ability of these systems to efficiently mobilize large gene cassettes makes them versatile for many genetic modification applications in these mosquito species.

Overall fitness impacts of the drive system are consistent with loads imposed by expression of the effector molecule transgenes and not insertion mutagenesis of the target site gene. Minimal larval effects in all hemizygous lines with greater adult effects are consistent with the activity of the transgenes. The enhanced effects in homozygous mosquitoes compared to their respective heterozygotes are also consistent with an expression load.

The effects on adult longevity and female reproductive output of the introduced TP24 and TP43 transgenes are mixed in *An. gambiae* and *An. coluzzii*. In particular, hemizygous AgTP24 males and females and AcTP24 males show significantly lower survival, while AcTP24 females are about the same as controls. In contrast, there is no significant effect on adult longevity of hemizygous AgTP43 males and females, but AcTP43 females show a significant reduction. The effects on adult longevity in homozygous AcTP24 and AcTP43 were also mixed, with significant effects seen in AcTP24 males and females and AcTP43 females, and a lesser effect in AcTP43 males. Significantly reduced female fecundity and fertility were seen in the hemizygous AgTP24 and AcTP43 strains and the homozygous AcTP43 strain. The combination of reduced adult longevity and female reproductive output could impact long-term drive dynamics. In particular, the AgTP24 and AgTP43 loads that prevented establishing homozygous strains are expected to have a profound negative impact on their sustainability, reminiscent of what was seen with the system that targeted the *cinnabar* gene ortholog in *An. stephensi* (Gantz et al. 2015; Bottino-Rojas et al. 2022). This load and resulting fitness costs preclude the *An. gambiae* strains from being considered for further development in the field.

As previously reported in TP13-based drive systems, the expression levels of effector genes assessed by RT-PCR differ in *An. gambiae* and *An. coluzzii* backgrounds (Carballar-Lejarazú et al. 2023). While the methods do not allow quantitative comparison of gene expression levels, the AcTP24 and AcTP43 effectors were expressed abundantly, not only in the designated compartment, the midgut, for all effectors except the fat body for m2A10, but also in ectopic compartments. The ddPCR analysis of the Try1/Rel2 component clearly shows a transgene-mediated increase in the abundance of the gene transcript.

**Fig. 6.**
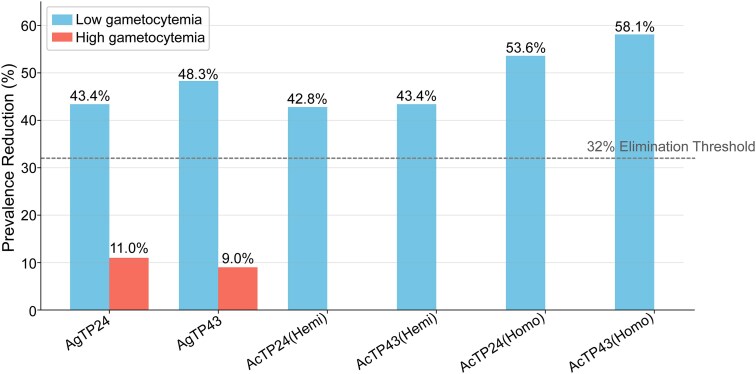
Impact of effector gene activity on *Plasmodium falciparum* oocyst prevalence in post-bloodmeal infected hemi- and homozygous AgTP24, AgTP43, AcTP24, and AcTP43 females. Percent reductions in oocyte prevalence following parasite challenges with low (blue) and high (orange) gametocytemia bloodmeals. The target 32% threshold for elimination predicted by Blagborough et al. (2013) is shown as a horizontal dashed line.

Quantifying and modeling human malaria parasite transmission requires consideration of several key parameters, including infection prevalence, mean infection intensity, the extrinsic incubation period (EIP) from gametocyte ingestion to sporozoite maturation, the frequency and duration of infectious bites, and ultimately, the impact on human incidence rates. These metrics are used widely in transmission models and form a basis for interpreting our findings (Grosso et al. 2025). The present study demonstrates that both hemi- and homozygous AgTP24, AgTP43, AcTP24, and AcTP43 lines are capable of significantly reducing *P. falciparum* oocyst and sporozoite burdens under laboratory challenge conditions. In low-gametocytemia, single-generation infections, representative of many naturally occurring transmission events, the prevalence reductions achieved by these lines were substantial. Notably, the strongest effects were observed in homozygous AcTP24 and AcTP43 females, with infection prevalence decreases approaching or exceeding 58% to 60%. These values far surpass the 32% oocyst reduction threshold predicted by Blagborough et al. (2013) to be necessary to achieve population-level interruption of malaria transmission (Fig. 6).

However, infection dynamics vary across ecological and epidemiological settings. While mean oocyst infection intensities in our controls ranged between 2.2 and ∼4, consistent with some field reports, other studies have documented considerably higher burdens (≥10 oocysts per midgut), particularly in high-transmission settings influenced by seasonal patterns, human infectious reservoirs, and mosquito biting densities (Bompard et al. 2020). Higher gametocytemia challenges in our assays produced elevated MIIs (∼60 oocysts in controls), which corresponded to smaller proportional reductions in infection prevalence, underscoring the influence of parasite exposure dose on intervention efficacy.

The data presented here are encouraging in terms of sporozoite threshold MIIs, which have been used to approximate potential transmission risk (<10,000 sporozoites per salivary gland pair per mosquito; Aleshnick et al. 2020; Graumans et al. 2020). None of the AgTP24, AgTP43, AcTP24, or AcTP43 mosquitoes in the low-gametocytemia treatments exceeded this limit. It is possible that the few mosquitoes retaining relatively high parasite counts result from suboptimal effector gene expression, potentially influenced by differential promoter activation due to variability in bloodmeals. The parasites also may represent pre-existing genetic variants that are not susceptible to the different modes of action of the tested effector molecules. However, this is not likely because the compound effector design is intended to reduce the probability of existing resistance. Work is in progress to directly assess whether the residual parasites are susceptible to the compound effector molecules.

It is also important to recognize that the parasite challenge assays rely on long-cultured laboratory *P. falciparum* strains and artificial membrane feeding, both of which may differ from natural host/vector/parasite interactions. Laboratory results may under- or overestimate performance in the field depending on genetic diversity, parasite adaptation, and vector population structure (van de Vegte-Bolmer et al. 2021). To address this, parasite challenge assays with recently-isolated and genetically-diverse *P. falciparum* strains are ongoing. It is encouraging that a recent report has shown that a laboratory-generated gene-drive system works well against patient-derived parasites in an *An. gambiae* strain (Habtewold et al. 2026). Finally, as with any gene drive-linked effector system, there is a possibility that prolonged circulation of the construct in a target population could lead to functional erosion via mutation or aberrant recombination. Extended population cage experiments are underway to assess long-term drive system stability and effector gene integrity and expression over a two-year, multi-generational period.

Overall, these findings support the conclusion that TP24 and TP43 compound effector gene cassettes have the potential to substantially reduce *P. falciparum* transmission, particularly under lower-intensity infection scenarios that more closely resemble typical field conditions. In particular, the strong suppression observed in homozygous AcTP24 and AcTP43 lines highlights their promise for integration into population-modification strategies, provided that their efficacy and stability are confirmed in genetically-diverse field populations of both mosquitoes and parasites.

## Supplementary Material

jkag014_Supplementary_Data

## Data Availability

The datasets generated and/or analyzed during the current study are available in the figures, tables, and Supplementary Materials. Plasmids and mosquito lines are available from the authors on request. Supplemental material available at G3 online.
