# Supplementary material for: Compound effector genes suppress malaria parasite infections in gene-drive population modification strains of the African malaria mosquitoes, Anopheles gambiae and Anopheles coluzzii
Source: G3 (Bethesda). 2026 Mar 3;16(8):jkag014. doi: 10.1093/g3journal/jkag014 (PMC13439948; doi:10.1093/g3journal/jkag014)
Supplement: Supplementary file 1 [file jkag014_supplementary_data.pdf]

SUPPLEMENTAL MATERIAL

Table S1. List of oligonucleotides and primers.

Table S2. *Anopheles gambiae* AgTP24 and AgTP43 prevalence, mean intensities of infection and statistical analyses.

Table S3. *Anopheles coluzzii* AcTP24 and AcTP43 prevalence, mean intensities of infection and statistical analyses.

Table S4. Plasmid component sequences.

References

Table S1. List of oligonucleotides and primers.		
**Primer Name**	**Sequence (5' – 3')**	**Ref.**
**TP334-CARDINAL-SWAP-1-F**	TTCCCCTCGTCCTTGCGACTAGTTAATTAAAGGGTTTTAGAGCTATGCT	1
**TP335-CARDINAL-SWAP-1-R**	AGCATAGCTCTAAAACCCTTTAATTAACTAGTCGCAAGGACGAGGGGAA	1
**TP336-CARDINAL-SWAP-2-F**	TTCCCCTCGTCCTTGCTGATTATGATCTAGAGTCGGTTTTAGAGCTATGCT	1
**TP337-CARDINAL-SWAP-2-R**	AGCATAGCTCTAAAACCGACTCTAGATCATAATCAGCAAGGACGAGGGGAA	1
**TP292**	GCCGCCTGTTTAAACCGCCGGCCCACATGGAATAA	1
**TP288**	GGTTGAAGTGTTTCAGTTTAAACAACAAAAATGAATAAATAAACAAGAACTGCATAA TAATGAAATGATC	1
**TP268**	TATTTATTCATTTTTGTTGTTTAAACTGAAACACTTCAACCACAACAG	1
**TP269**	AGACAAACGGATAAACAAGATCGACGGTCGACCGGAAATGGTA	1
**TP270**	TACCATTTCCGGTCGACCGTCGATCTTGTTTATCCGTTTGTCT	1
**TP271**	ATCAGTATCAGAGTTTCAGGATCAGACGCGGAGCTCGCCCGGGGATCT	1
**TP276**	GTATCAGAGTTTCAGGATCAGACGCGGAGCTCGCCCGGGGATCTAA	1
**TP277**	ATGGCTGATTATGATCTAGAGCTACAGGAACAGGTGGTGGCGG	1
**TP399**	TCGAGCACCACCACCACCACCACTAAATCGAACGAACACCAATGGAATGGCAATGGA A	1
**TP402**	GCTATGGCGGCCGCCTGTTTAAACCGATCTTGTTTATCCGTTTGTCTCG	1
**TP396**	GCCACCGCCAGGATGGCCATGATGCTAGATTTGATTGGGT	1
**TP403**	GATTGTGTTTTACGCGGTTTAAACCTGATCCTGAAACTCTGATACTGATCCC	1
**TP397**	ACCCAATCAAATCTAGCATCATGGCCATCCTGGCGGTGGC	1
**TP398**	TTGGTGTTCGTTCGATTTAGTGGTGGTGGTGGTGGTGCTCGAGCGTAG	1
**TP394**	AGTTTCAGGATCAGGTTTAAACCGCGTAAAACACAATCAAGTATGAG	1
**TP395**	ATTATGATCTAGAGGTTTTTACTTGTACAGCTCGTCCATGCCGCC	1
**TP280**	GCTCCACGATCAGTATGGATTCAG	1
**CO53**	CGGGAATTAGCAATGTTTTACA	1
**TP180**	GATTCCGGATCGACGATGCG	1
**TP149**	AGCGAGCCGGTACTGTTCAA	1
**VGSEQF4**	GAAGCCATACCATGAGTCCG	2
**TP471**	GCTGGACTGGACTGGTCG	1
**TP406**	CGGCTGGATTAACGAAGAGAAG	1
**AK5**	GTACTCGTACGGTCGCTCCTTA	2
**AK6**	ATTGTTGTTGCAGATGAGTCGT	2
**1C3-RTPCR-F**	TACGTTTAAGACGTACGCCATGAACTG	2
**1C3-RTPCR-R**	CTTCAGCTCCAGTTTGGTACCCG	2
**2A10-RTPCR-F**	GAAGACGTAAGGGAAGCGGC	2
**2A10-RTPCR-R**	GGATTAACACGATCACGGAAGAACC	2
**TB367**	GGTGCCTCGATGAAGATCTC	1
**TP371**	CCACACATCGAAGTACACGT	1
**TP35**	CGCCCGTTTGATCTCCAGT	1
**TP374**	GGCTTCACCTTCAGCGATTA	1
**TP148**	GCAGTGGTCAGTGTTGGAGAG	1
**TP150**	CATATTGTTGATTGGGTTCGA	1
**RPS7-F**	GACGGATCCCAGCTGATAAA	3
**RPS7-R**	GTTCTCTGGGAATTCGAACG	3
**RT-MULTIE-F**	GGCATCGGCGCCGTGCTGAAG	3
**RT-MULTIE-R2**	CTTGTCGTCATCGTCTTTGTAGTC	1
**TP364**	TACTCGTCCAGCTCGTAGAA	1
**TP365**	CGGTCTACTCGAACCCAATC	1
**REL2-DDPCR-HEX**	[BHQ1]TCGCCACCGTCCACCCCGCT[HEX]	1

1. This study.
2. Carballar *et al.*, 2023.
3. Dong *et al.*, 2020.

Table S2. ***Anopheles gambiae*** **AgTP24 and AgTP43 prevalence, mean intensities of infection and statistical analyses.**

	**Oocysts/midgut: high infection**				**Oocysts/midgut: low infection**			**sporozoites/salivary gland: low infection**		
	**WT-G3[a]**	**WT-X1[a]**	**TP24-Hemi**	**TP43-Hemi**	**WT-X1[a]**	**TP24-Hemi**	**TP43-Hemi**	**WT-X1[a]**	**TP24-Hemi**	**TP43-Hemi**
n	63	62	51	56	117	85	103	76	72	72
MII	60.3	68.0	31.2	29.5	2.2	0.7	0.7	3112.5	664.6	381.8
median	60	63.5	32	32.5	2	0	0	1612.5	0	0
Mann-Whitney P-value	0.4929 (ns)	Ctl	<0.0001	<0.0001	Ctl	<0.0001	<0.0001	Ctl	<0.0001	<0.0001
Prevalence	100.0%	100.0%	88.2%	91.1%	76.9%	43.5%	39.8%	75.0%	43.1%	34.7%
Fisher's exact P-value	>0.9999 (ns)	Ctl	0.0003	0.0032	Ctl	<0.0001	<0.0001	Ctl	<0.0001	<0.0001
range	17-162	9-169	0-87	0-89	0-14	0-4	0-6	0-23475	0-4350	0-2325
% reduction[b]	-	-	49.6%	48.8%	-	68.4%	68.2%	-	78.6%	87.7%

[a]Control samples: WT-G3, WT-X1.
[b]Percent reduction in the MII.
Abbreviations: Ctl, control; Hemi, hemizygous; MII, mean intensities of infection; n, number of mosquitoes; ns, not significant.

Table S3. ***Anopheles coluzzii*** **AcTP24 and AcTP43 prevalence, mean intensities of infection and statistical analyses.**

	**Oocysts/midgut low infection**					**sporozoites/salivary glands low infection**				
	**Ctl-X1[a]**	**TP24-Hemi**	**TP43-Hemi**	**TP24-Homo**	**TP43-Homo**	**Ctl -X1[a]**	**TP24-Hemi**	**TP43-Hemi**	**TP24-Homo**	**TP43-Homo**
n	107	71	79	70	84	79	77	75	69	69
MII	4.0	1.9	1.5	0.6	0.5	3379.4	968.1	918.3	443.6	342.9
median	2	0	0	0	0	1335	0	0	0	0
Mann-Whitney P-value	-	<0.0001	<0.0001	<0.0001	<0.0001	-	<0.0001	<0.0001	<0.0001	<0.0001
Prevalence	73.8%	42.3%	41.8%	34.3%	31.0%	72.2%	41.6%	38.7%	30.4%	29.0%
Fisher's exact P-value	-	<0.0001	<0.0001	<0.0001	<0.0001	-	<0.0001	<0.0001	<0.0001	<0.0001
range	0-24	0-16	0-17	0-8	0-9	0-24000	0-8900	0-10240	0-4800	0-4500
% reduction[b]	-	52.9%	61.5%	83.9%	86.5%	-	71.4%	72.8%	86.9%	89.9%

[a]Control samples: WT-G3, WT-X1.
[b]Percent reduction in the MII.
Abbreviations: Ctl, control; Hemi, hemizygous; MII, mean intensities of infection; n, number of mosquitoes; ns, not significant.

Table S4. Plasmid component sequences.	
**Feature**	**Sequence (5' – 3')**
**pTB31-AgU6-gRNA-template**	
**AgU6-gRNA template**	TGTGGCGCGCCCGTAGAGACATTTTGTACCAGAATATTTAAGTAAAATAAACATTATTTTTACCGGAATGTATACATAATTACGAAAA<br>ATGTTTTATTTTTCAAAAAAAATGTTCAAATTTCTGCCATAATGGTATTTGCTTGAAATCTAAACCCGAGCACACACTTTCTTGCTCC<br>GTGCTCTCACCCTTTGCCGGCTCTTTCTTCTCTTTCTCACTGCGATACGAACCCTACGGTACGCGACGTTCGGCTGGGAATGAGTTCC<br>AGCATTAGATGGTGAGTGCTTGTTGGCCACATATACACTGAAGCGGTTTGGCTCTTGGTTATTCTAGAAAACTCTGTTTTTGAATTAA<br>TCATAGGAAAACTGTTTATTCTTTTTTGATATTTCAACGTATTACAATGAATAGTAACTTTTGATTAAAAAAGAGATGAAGCAATACA<br>AGAAATGTTGAAAAGTTTATGAAACATCACATTAGCGTGAGTTACGGCAGGATCAAAACCTTATAACAGTCACACTCAGGCAAAAAAT<br>CCTTCTTGAATATCCTTTATGCATCGCTAGAGCAAGGATTGAAAGCGCAAAGTATATATACAACCTTTTTTCCCCTCGTCCTTGGGGT<br>CTTCGAGAAGACCTGTTTTAGAGCTAtgctgGAAAcagcaTAGCAAGTTAAAATAAGGCTAGTCCGTTATCAACTTGAAAAAGTGGCA<br>CCGAGTCGGTGCTTTTTTTGTCAAATGTTTTTCTTATTTTCATCATTGGGGACTAGAACCGTATAATATATTTTAATTCTTTTTCATG<br>AAAGATATCACATTCATTAACCGAACAAAGAGAGAGAGAGAGAGAGAAATAGATAGAGAGAGAGAGAGAGAGAGAGAGAGAGAGAG<br>AGAGAGAGAGAGAGAATATTGTTGTTCGATACAGAAAATGAGTACGTAGCAGTTATTTGGATATGATTTAGAATCTCAAAATTCCCCA<br>AAACTGTTCTCAAGGTATTCAAATAAGTTTGTTCAAATAAACGACAGCTTTATTTATTCTTGATCACCGTGCAATGTTCTTTCGCAAC<br>CTTTAGCTGAGTTTGTTTCATTTATATTTTGTTCGTTTTCATTGCCTTAAGTAG
**pTB32-AgU6-gRNA-Swap-1**	
**AgU6-gRNA-swap-1**	TGTGGCGCGCCCGTAGAGACATTTTGTACCAGAATATTTAAGTAAAATAAACATTATTTTTACCGGAATGTATACATAATTACGAAAA<br>ATGTTTTATTTTTCAAAAAAAATGTTCAAATTTCTGCCATAATGGTATTTGCTTGAAATCTAAACCCGAGCACACACTTTCTTGCTCC<br>GTGCTCTCACCCTTTGCCGGCTCTTTCTTCTCTTTCTCACTGCGATACGAACCCTACGGTACGCGACGTTCGGCTGGGAATGAGTTCC<br>AGCATTAGATGGTGAGTGCTTGTTGGCCACATATACACTGAAGCGGTTTGGCTCTTGGTTATTCTAGAAAACTCTGTTTTTGAATTAA<br>TCATAGGAAAACTGTTTATTCTTTTTTGATATTTCAACGTATTACAATGAATAGTAACTTTTGATTAAAAAAGAGATGAAGCAATACA<br>AGAAATGTTGAAAAGTTTATGAAACATCACATTAGCGTGAGTTACGGCAGGATCAAAACCTTATAACAGTCACACTCAGGCAAAAAAT<br>CCTTCTTGAATATCCTTTATGCATCGCTAGAGCAAGGATTGAAAGCGCAAAGTATATATACAACCTTTTTTCCCCTCGTCCTT**GCGAC**<br>**TAGttaattaaAGG**GTTTTAGAGCTAtgctgGAAAcagcaTAGCAAGTTAAAATAAGGCTAGTCCGTTATCAACTTGAAAAAGTGGCA<br>CCGAGTCGGTGCTTTTTTTGTCAAATGTTTTTCTTATTTTCATCATTGGGGACTAGAACCGTATAATATATTTTAATTCTTTTTCATG<br>AAAGATATCACATTCATTAACCGAACAAAGAGAGAGAGAGAGAGAGAAATAGATAGAGAGAGAGAGAGAGAGAGAGAGAGAGAGAG<br>AGAGAGAGAGAGAGAATATTGTTGTTCGATACAGAAAATGAGTACGTAGCAGTTATTTGGATATGATTTAGAATCTCAAAATTCCCCA<br>AAACTGTTCTCAAGGTATTCAAATAAGTTTGTTCAAATAAACGACAGCTTTATTTATTCTTGATCACCGTGCAATGTTCTTTCGCAAC<br>CTTTAGCTGAGTTTGTTTCATTTATATTTTGTTCGTTTTCATTGCCTTAAGTAG
**pTB33-AgU6-gRNA-Swap-2**	
**AgU6-gRNA-swap-2**	TGTGGCGCGCCCGTAGAGACATTTTGTACCAGAATATTTAAGTAAAATAAACATTATTTTTACCGGAATGTATACATAATTACGAAAA<br>ATGTTTTATTTTTCAAAAAAAATGTTCAAATTTCTGCCATAATGGTATTTGCTTGAAATCTAAACCCGAGCACACACTTTCTTGCTCC<br>GTGCTCTCACCCTTTGCCGGCTCTTTCTTCTCTTTCTCACTGCGATACGAACCCTACGGTACGCGACGTTCGGCTGGGAATGAGTTCC<br>AGCATTAGATGGTGAGTGCTTGTTGGCCACATATACACTGAAGCGGTTTGGCTCTTGGTTATTCTAGAAAACTCTGTTTTTGAATTAA<br>TCATAGGAAAACTGTTTATTCTTTTTTGATATTTCAACGTATTACAATGAATAGTAACTTTTGATTAAAAAAGAGATGAAGCAATACA<br>AGAAATGTTGAAAAGTTTATGAAACATCACATTAGCGTGAGTTACGGCAGGATCAAAACCTTATAACAGTCACACTCAGGCAAAAAAT<br>CCTTCTTGAATATCCTTTATGCATCGCTAGAGCAAGGATTGAAAGCGCAAAGTATATATACAACCTTTTTTCCCCTCGTCCTT**GCTGA**<br>**TTATGATCTAGAGTCG**GTTTTAGAGCTAtgctgGAAAcagcaTAGCAAGTTAAAATAAGGCTAGTCCGTTATCAACTTGAAAAAGTGG<br>CACCGAGTCGGTGCTTTTTTTGTCAAATGTTTTTCTTATTTTCATCATTGGGGACTAGAACCGTATAATATATTTTAATTCTTTTTCA<br>TGAAAGATATCACATTCATTAACCGAACAAAGAGAGAGAGAGAGAGAGAAATAGATAGAGAGAGAGAGAGAGAGAGAGAGAGAGAGAG<br>AGAGAGAGAGAGAGAGAATATTGTTGTTCGATACAGAAAATGAGTACGTAGCAGTTATTTGGATATGATTTAGAATCTCAAAATTCCC<br>CAAAACTGTTCTCAAGGTATTCAAATAAGTTTGTTCAAATAAACGACAGCTTTATTTATTCTTGATCACCGTGCAATGTTCTTTCGCA<br>ACCTTTAGCTGAGTTTGTTTCATTTATATTTTGTTCGTTTTCATTGCCTTAAGTAG
**pTP20-Swap-in-pTP13-HA**	
**HA1 SWAP (same as Ag-card HM1)**	CTGGAACAGCCGCTCGTCGTCCCAGTGCGGGTTTGCGCGGGCCAGCCCGCGGGCAAGGCTGTTGTGGTGCCGGGCCCAGATCAGATGC<br>ATCGAGGTGAGATGCAGATTCTCGTTCGCCCGCGCATCGCCCGACTCGAAGCAGTACTTGCCGGCCGCATTCATCTCCTGCTCGTTGC<br>ACCCGTCGAGCGGATCGGTCGACACGGGCAGCAGATCGCGACCGTCCGGCGTGCGCAGCATACGCAGCTGGCCACCGGCGCCCGTGCG<br>CAGCGCGCCCATACGCTCGTCGTCCGAGCCGTACACGACCGAGCCGTCGATGAAGGCGGTCGCCTGGTTCAGCTGCTGCCGCGGACCG<br>AAGTGACCGGTCGGGGCCGGCACCGAGCGCACAAAGTTCATGCAGGTCACGTTGTACTGGGTGTAGTACGGGTCGCCCGGACCGAGCG<br>GCACGGGAAAGCACTCGGGATGTTGCGGCTGCCCCGGATCGCAGCACTCGATCGGCTTCCCGTCCACGCCCTGGTTCAGCGCGGTGGA<br>GGTTATGTCGTGATCGAGAAACTGGCCCCACACCGCCAGCATCACGCTAAAGTTCGGATCGTTGTGGTACGATGGCGATGGATTTCG<br>AGCGACACCTGCCGGGCGCTGGGCAGTTCGGCCCCATCGACGGAGGCACGCGGTGCCGATATGCCATCGCCATAGTCCGGGTTGAGCT<br>GGCGCCGGAACGGTATCATCGCGACGCCGTACTCGTACGGTCGCTCCTTACTGTTGCACGTCCCGTTGGCCGTGCGATAGCGCGCGTT<br>AAAGTCACAGCGCGGTTGACGGTGCGCACGCGGCAAATCCATCGGTGGGCCGCGCCCGATCGAGCCCCGACGACGGATGTCCAACCGG<br>CGCACCAGTGCCTGCGTGGCACGATCCTCCACGAAGCCGACCTTTGCCGCCAGCCGGGCCGCTTTCGTTGCACCGACGGACTTTTGGT<br>GGCGGAAGGAGGGCGAGTTAAGCGGGGGCGACGAGAGCGTCTCCTCCAGCAGCTCCTTATCGCCCAGCGCCTTCTCGCCATCCGCTAT<br>GGCGGCCGCCT
**HA2 SWAP**	CTCTAGATCATAATCAGCCATACCACATTTGTAGAGGTTTTACTTGCTTTAAAAAACCTCCCACACCTCCCCCTGAACCTGAAACATA<br>AAATGAATGCAATTGTTGTTGTTAACTTGTTTATTGCAGCTTATAATGGTTACAAATAAAGCAATAGCATCACAAATTTCACAAATAA<br>AGCATTTTTTTCACTGCATTCTAGTTGTGGTTTGTCCAAACTCATCAATGTATCTTAAAGCTTATCGATACGCGTACGGCGCGCCggc<br>cgtgtggtttaacccaccaaactggcagtttcttggctttgttcgatgcaactggatttttagtacaggaaattggtggcgttttcgg<br>tatcttggttatttgggtagtgtatagtggtgttttcgaaattcatttttgttcattaacagttgttcaacctatagttatttattac<br>aataatattctactaacgattaaccgattgattcaaagtgaataaattataaaactagtgatttttttTaaatttttgtatgatttt<br>gtcatttcttggatcattatccgtgcaaatcgattaaccggcaacagtccggtcccgagctgcccgaataatcgacgttctactgtat<br>tcctaccgactttttgatatgcctactgactcaccgatgagccccctaaaactaccgatttttatttatcctaccgaaaatcacaga<br>ttttttcgtaatactgaccaaaaagtcatgtaatcatttcccaaatcacttaatgtattaaactccatatggaaatcactagcaacca<br>gaacaagaagttcaacagagacaacccccgtgtacttcatgagatgcgatgatatcggacgcgctggtaaaattttatatggtatttg<br>aaagatactgtaagacgtgcgatttttaatacgatggaatcaattcaacagtcaattatgaattaattaagaatcaatttgtagaaa<br>aaatctctaatgtatgttttgttatcgtaacagttaccaattcatccaaataatcgacatagcgacacagcacattcgatttagcaat<br>gtcctaagcggccgcgtggagccccaaaaaaagggagtccgcaacgcttagaagcaaatgtgcatctcaatcattcgttggg
**pTP19-TRY1-Rel2**	

**TRY1-3'UTR**	CGCCGGCCCACATGGAATAAAACGTAAGCAAACTTTGACAGCTAGTCGAGCAGTTGGCGCTGACAGCGAAATTGTCGAGCTCGAATGG<br>AACTGGCGGAACGGACGGAATATTCGTGGAAGTGAATTTGAAGAAGTTTATTCTTCATAAGAACCAAAAATACAAATTTATAACTAA<br>ATCGTGTTTCTTTACGCCTGGATAAATGTAAAAATGTcTAaTctGAAGATGTGTTGGCACCTCCAACGTTGACAGAGTTTCGATTCCC<br>AGCGAGGTCGTCTTCCCGTAGTGAAGACTGGCCATCCTTTCGCGTGATAGTAAACTATCTAGTAGGAATTACTTTTCTCCTTATTGAA<br>TTCAATTAACCCATCTTTTATGCGTTTTGAT
**Rel2 CDS**	ttaTTCCGAGTTACAGGGGAAGTCGCTCGAGTCGAACGCTACGTCCGACGCGATCGTCGTCGGAATGTTCAGTATCTCCTGGATGATG<br>CTGGCCTTCGTTAGCTCCTTAGAGATGGTGGTGGTGGAACCGATGCCGCTCATAATGTCCGGATGATGGGCTGACGTCGTAAGCGATG<br>TGGTCGACGTTGGTTCTTGACCTTCCGGAATGGCACCGCCGCCAGCATGATTGGCCAGCATTGGGAATGGTTGATGCAGGGGCGGTAG<br>TCGTGTAGTTGATCCTGAACCGATAACACCACCACCACCACCACCTCCTCCTCCACCTCCACCACCACCTACTCCAGCGGGTCCAACA<br>GGCCCGTTCGGGTCGTTCTGGATGACGGTGGGAATTTCGGACGACACATTGCCCGAGCTGATGCGGAGCCGCTTGCGCGACGCACCAC<br>TAGCACCAGCGCCCGGCACCACCATCATGCCGGGCCGCGGTTTGTACTTGAACAGTACCGGCTCGCTCTGGCACCGGTCGCGCGGCCG<br>GAACAGCTGCATCAGCACCTCGACCGGCTCGGTAATGTCCTTGTGCCGGTACGGTGGCGTCTTGAACGCGATCGCATACTGGTGATGG<br>ACGTCCGCCTCGGAAAAGATGGCCATCTCCTGCCACACCTCCTGGTCGTACTCGTCCAGCTCGTAGAACCGAATCTTGATGTTATTTT<br>TGCACACCTTTTCGACAAACATAAACACCTCCTCGCCACCGTCCACCCCGCTGACGGTGGTGCTGAGCCGGCAGATCTTCAGCTCGCC<br>CGTAAGTGCACTTTTCATATTGTTGATTGGGTTCGAGTAGACCGGCTCACAGATCGGGACCCAGCGGCCCGTACCCGGCTCCACCCGG<br>TACGCCCGGAAGCACAGGCACACCTGATTGAGGTTCATCGTGCGCGCCATTACGGCCGCCTCCTTCTGCATCTGCTGCTCCTCGCGCT<br>CGGTCGGCTCCCGGTTCAGCTCGCACAGCCGATGCTTGCGCAGCTTCTTGTACAGCTCCTCGGCGATGAACTTCTTCGCCGTGTGTAT<br>GATGCCCATACCCTGGAAGGTGGCCACATACTTGCCGTCCCCGCCAACACCCTCCGATGGTTCAGCCGCTCCCGCCGCGCCACCCACA<br>TCCAGATCGTGCGGATCGATCAGATCCAGCTCGCCGGACTTGATCACCAGATGGTGCGAATGGGGGGCGCGCCGTTGCGGATCCACCT<br>GATAGAGGGAGCAGCGCACCTTCGCCTCCCCACCGTACCCACGCAGCTCGACGGTCGGGAACGTTTTCTTCGACTTCTCCGTCCGGCT<br>GCCCATCAGCGAACCGTGCGTCCCGTGCATCTCCGACTGGTAGCGGAAGCGAAACTTGTCCACCGGCTGCTCCAGGATCACCAGATGC<br>GGCTCGCTGTAGTTCGGTTCCGGTGCGGCAAACGCTTCGTTCGACGATGTGACGTAGGTGAAGGTTTGCGTTTGCAGTTGCTCCTGGT<br>CGAGATTCAGCAGCTGCGGTGTGTAATAGTGATGCTGCTGCTGTTGAAGTTGTTGATGTTGATGaTGTTGTTGCTGCTGTTGCTGCTG<br>CTGCTGCTGCTGCTGATAACCCATGCCAGGGTAGTTCATCGTGCCGGCGCTACTGGAAAGGTTCAGGTTTTGCAGCGTATAGGCAGCA<br>CTCTGATCGCTGGCACTGGACTCCGGGGACATGTTGCTGGCCCGGCTGTTGGGCGATGCGACCGACGACGGTGACGATGGCGATGCGG<br>ACGATGACGATGGCGAAAGCGCACTGGCATAGGTGCGACCCGGGCCGAGCGTAAACTCTCCAACACTGACCACTGCcat
**TRY1-5'UTR**	CGTGTCCGGCACTCGTATGATCGATGGGAATGTAGATGCCAACTATATATACAGTGCGGTCAGTCCAGgTTCCGTGGCAGTGAACTTG<br>TCCCGTTCGGCGGTATTAATTGATAGCGATATCAATTGCTGATCGcCCGAACCCACTCAGACAATTTCTCGGTTCAATGGTGTACTTA<br>AgATGAACACGATAACACTACGGTACAATCTCTCCACTTAACATtGaCTATCGTATAAGACGtCCGTAACTGAGGATGGACAGaAAAC<br>CCTCCGACGCACCTGAAAGGCACTTGAAGAGTGTTTATCGTGTAGTGCAAATATAGGATGTCTGACCACTATCAGGCACTTGGTGGC<br>AATAATCCCGTGTCTTGTCGGGATCTCCACTATTTTGGACAACTTTAGTCATcCTACATGTTTACTTTTAGGATTAGACGCATTAGAA<br>CAAAGGGATTATTGTCGGCATTGCGGGTTTttcTTCTGAAAGTACAAGCCTCTCTCGAATGGTCGTGCGACAGTTTTGAACAGTTCCA<br>agCTAAAGTGGGaGGCTTTTAACGGAATGGAAtGGATTTCCAGTACCTGGTTCCAaCCATaGATCCTgCAAGAcATTGTTTAGtTATA<br>tgAAGATCGGCCTCTGATGGTATTgGATcTTAAATATGATGAATTTCTGTTGaTTAAAcTctAGtTAAAGCTGAACGAGCGATCCCGT<br>GGTAtATTCGTTAACTTgTGCGACaCAACAcCAaGCCCGTTATGGGTTCAAGCTCCGAAACAtGACCCTAcCTCTTATaCGTaAGACT<br>GACTCTCCAGTCCATAGATAATCAATGCCATGTTTATTaATAGTTAAGACgTACTACAGCTGTTaCGAAGAAGAATCAAGtTGAATGG<br>AATATTCTaACATCATTTGATCTACGAGACTaAagCTACCaCGTTTATCAGATATCCAATTCTTTGGTGCAAGCTCACACGAtTGCCc<br>ATGCTCGAGAAAATGTTCCTATGTTCTTTCCTTGTAAGTGCTATTTATCTCTCAATTTCtTTGTATCAAAAgATCATTTcATTATTA<br>TGCAGTTCTTGTTTATTTATTCATTTTTGTt

pTP24-SWAP-Try1_Rel2-AgPer_1B2-G12_m4B7-3xP3_dsRed

**HA1 SWAP (same as Ag-card HM1)**	CTGGAACAGCCGCTCGTCGTCCCAGTGCGGGTTTGCGCGGGCCAGCCCGCGGGCAAGGCTGTTGTGGTGCCGGGCCCAGATCAGATGC<br>ATCGAGGTGAGATGCAGATTCTCGTTCGCCCGCGCATCGCCCGACTCGAAGCAGTACTTGCCGGCCGCATTCATCTCCTGCTCGTTGC<br>ACCCGTCGAGCGGATCGGTCGACACGGGCAGCAGATCGCGACCGTCCGGCGTGCGCAGCATACGCAGCTGGCCACCGGCGCCCGTGCG<br>CAGCGCGCCCATACGCTCGTCGTCCGAGCCGTACACGACCGAGCCGTCGATGAAGGCGGTCGCCTGGTTCAGCTGCTGCCGCGGACCG<br>AAGTGACCGGTCGGGGCCGGCACCGAGCGCACAAAGTTCATGCAGGTCACGTTGTACTGGGTGTAGTACGGGTCGCCCGGACCGAGCG<br>GCACGGGAAAGCACTCGGGATGTTGCGGCTGCCCCGGATCGCAGCACTCGATCGGCTTCCCGTCCACGCCCTGGTTCAGCGCGGTGGA<br>GGTTATGTCGTGATCGAGAAACTGGCCCCACACCGCCAGCATCACGCTAAAGTTCGGATCGTTGTGGTACGATGGCGATGGATTTCG<br>AGCGACACCTGCCGGGCGCTGGGCAGTTCGGCCCCATCGACGGAGGCACGCGGTGCCGATATGCCATCGCCATAGTCCGGGTTGAGCT<br>GGCGCCGGAACGGTATCATCGCGACGCCGTACTCGTACGGTCGCTCCTTACTGTTGCACGTCCCGTTGGCCGTGCGATAGCGCGCGTT<br>AAAGTCACAGCGCGGTTGACGGTGCGCACGCGGCAAATCCATCGGTGGGCCGCGCCCGATCGAGCCCCGACGACGGATGTCCAACCGG<br>CGCACCAGTGCCTGCGTGGCACGATCCTCCACGAAGCCGACCTTTGCCGCCAGCCGGGCCGCTTTCGTTGCACCGACGGACTTTTGGT<br>GGCGGAAGGAGGGCGAGTTAAGCGGGGGCGACGAGAGCGTCTCCTCCAGCAGCTCCTTATCGCCCAGCGCCTTCTCGCCATCCGCTAT<br>GGCGGCCGCCT
**TRY1-3'UTR**	CGCCGGCCCACATGGAATAAAACGTAAGCAAACTTTGACAGCTAGTCGAGCAGTTGGCGCTGACAGCGAAATTGTCGAGCTCGAATGG<br>AACTGGCGGAACGGACGGAATATTCGTGGAAGTGAATTTGAAGAAGTTTATTCTTCATAAGAACCAAAAATACAAATTTATAACTAA<br>ATCGTGTTTCTTTACGCCTGGATAAATGTAAAAATGTcTAaTctGAAGATGTGTTGGCACCTCCAACGTTGACAGAGTTTCGATTCCC<br>AGCGAGGTCGTCTTCCCGTAGTGAAGACTGGCCATCCTTTCGCGTGATAGTAAACTATCTAGTAGGAATTACTTTTCTCCTTATTGAA<br>TTCAATTAACCCATCTTTTATGCGTTTTGAT
**Rel2 CDS**	ttaTTCCGAGTTACAGGGGAAGTCGCTCGAGTCGAACGCTACGTCCGACGCGATCGTCGTCGGAATGTTCAGTATCTCCTGGATGATG<br>CTGGCCTTCGTTAGCTCCTTAGAGATGGTGGTGGTGGAACCGATGCCGCTCATAATGTCCGGATGATGGGCTGACGTCGTAAGCGATG<br>TGGTCGACGTTGGTTCTTGACCTTCCGGAATGGCACCGCCGCCAGCATGATTGGCCAGCATTGGGAATGGTTGATGCAGGGGCGGTAG<br>TCGTGTAGTTGATCCTGAACCGATAACACCACCACCACCACCACCTCCTCCTCCACCTCCACCACCACCTACTCCAGCGGGTCCAACA<br>GGCCCGTTCGGGTCGTTCTGGATGACGGTGGGAATTTCGGACGACACATTGCCCGAGCTGATGCGGAGCCGCTTGCGCGACGCACCAC<br>TAGCACCAGCGCCCGGCACCACCATCATGCCGGGCCGCGGTTTGTACTTGAACAGTACCGGCTCGCTCTGGCACCGGTCGCGCGGCCG<br>GAACAGCTGCATCAGCACCTCGACCGGCTCGGTAATGTCCTTGTGCCGGTACGGTGGCGTCTTGAACGCGATCGCATACTGGTGATGG<br>ACGTCCGCCTCGGAAAAGATGGCCATCTCCTGCCACACCTCCTGGTCGTACTCGTCCAGCTCGTAGAACCGAATCTTGATGTTATTTT<br>TGCACACCTTTTCGACAAACATAAACACCTCCTCGCCACCGTCCACCCCGCTGACGGTGGTGCTGAGCCGGCAGATCTTCAGCTCGCC<br>CGTAAGTGCACTTTTCATATTGTTGATTGGGTTCGAGTAGACCGGCTCACAGATCGGGACCCAGCGGCCCGTACCCGGCTCCACCCGG<br>TACGCCCGGAAGCACAGGCACACCTGATTGAGGTTCATCGTGCGCGCCATTACGGCCGCCTCCTTCTGCATCTGCTGCTCCTCGCGCT<br>CGGTCGGCTCCCGGTTCAGCTCGCACAGCCGATGCTTGCGCAGCTTCTTGTACAGCTCCTCGGCGATGAACTTCTTCGCCGTGTGTAT<br>GATGCCCATACCCTGGAAGGTGGCCACATACTTGCCGTCCCCGCCAACACCCTCCGATGGTTCAGCCGCTCCCGCCGCGCCACCCACA<br>TCCAGATCGTGCGGATCGATCAGATCCAGCTCGCCGGACTTGATCACCAGATGGTGCGAATGGGGGGCGCGCCGTTGCGGATCCACCT<br>GATAGAGGGAGCAGCGCACCTTCGCCTCCCCACCGTACCCACGCAGCTCGACGGTCGGGAACGTTTTCTTCGACTTCTCCGTCCGGCT<br>GCCCATCAGCGAACCGTGCGTCCCGTGCATCTCCGACTGGTAGCGGAAGCGAAACTTGTCCACCGGCTGCTCCAGGATCACCAGATGC<br>GGCTCGCTGTAGTTCGGTTCCGGTGCGGCAAACGCTTCGTTCGACGATGTGACGTAGGTGAAGGTTTGCGTTTGCAGTTGCTCCTGGT<br>CGAGATTCAGCAGCTGCGGTGTGTAATAGTGATGCTGCTGCTGTTGAAGTTGTTGATGTTGATGaTGTTGTTGCTGCTGTTGCTGCTG<br>CTGCTGCTGCTGCTGATAACCCATGCCAGGGTAGTTCATCGTGCCGGCGCTACTGGAAAGGTTCAGGTTTTGCAGCGTATAGGCAGCA

	CTCTGATCGCTGGCACTGGACTCCGGGGACATGTTGCTGGCCCGGCTGTTGGGCGATGCGACCGACGACGGTGACGATGGCGATGCGG<br>ACGATGACGATGGCGAAAGCGCACTGGCATAGGTGCGACCCGGGCCGAGCGTAAACTCTCCAACACTGACCACTGCcat
**TRY1-5'UTR**	CGTGTCCGGCACTCGTATGATCGATGGGAATGTAGATGCCAACTATATATACAGTGCGGTCAGTCCAGgTTCCGTGGCAGTGAACTTG<br>TCCCGTTCGGCGGTATTAATTGATAGCGATATCAATTGCTGATCGcCCGAACCCACTCAGACAATTTCTCGGTTCAATGGTGTACTTA<br>AgATGAACACGATAACACTACGGTACAATCTCTCCACTTAACATtGaCTATCGTATAAGACGtCCGTAACTGAGGATGGACAGaAAAC<br>CCTCCGACGCACCTGAAAAGGCACTTGAAGAGTGTTTATCGTGTAGTGCAAATATAGGATGTCTGACCACTATCAGGCACTTGGTGGC<br>AATAATCCCGTGTCTTGTCGGGATCTCCACTATTTTGGACAACTTTAGTCATcCTACATGTTTACTTTTAGGATTAGACGCATTAGAA<br>CAAAGGGATTATTGTCGGCATTGCGGGTTTttcTTCTGAAAGTACAAGCCTCTCTCGAATGGTCGTGCGACAGTTTTGAACAGTTCCA<br>agCTAAAGTGGGaGGCTTTTAACGGAATGGAAtGGATTTCCAGTACCTGGTTCCAaCCATaGATCCTgCAAGAcATTGTTTAGtTATA<br>tgAAGATCGGCCTCTGATGGTATTgGATcTTAAATATGATGAATTTCTGTTGaTTAAAcTctAGtTAAAGCTGAACGAGCGATCCCGT<br>GGTAtATTCGTTAACTTgTGCGACaCAACAcCAaGCCCGTTATGGGTTCAAGCTCCGAAACAtGACCCTAcCTCTTATaCGTaAGACT<br>GACTCTCCAGTCCATAGATAATCAATGCCATGTTTATTaATAGTTAAGACgTACTACAGCTGTTaCGAAGAAGAATCAAGtTGAATGG<br>AATATTCTaACATCATTTGATCTACGAGACTaAagCTACCaCGTTTATCAGATATCCAATTCTTTGGTGCAAGCTCACACGAtTGCCc<br>ATGCTCGAGAAAATGTTCCTATGTTCTTTCCTTGTAAGTGCTATTTATCTCTCAATTTCtTTGTATCAAAAAgATCATTTcATTATTA<br>TGCAGTTCTTGTTTATTTATTCATTTTTGTt
**AgPerProm**	TGAAACACTTCAACCACAACAGACGCTGATAACAAGACGACGGTGTGCCGATCCGAGACGGCGCCCGTGGAGCGGCGTGAAGCTGAGGG<br>AAGCGAACCTTCCTTCTGAATTGTGATTTGTTTCCTGCACTTTTTGCACAGCTGCAGCTTTATATGCCATTTACATCTCGGTGCAAAT<br>AAAAGAGCGCCTCAGTTAGCCTTCCACTAATCCACTAATGCAGCATGCAAGCTTACAACTGGGGCTCGTAAACGGAACCAACACACAT<br>gCTCCGTTCCCGCCCGTCGTCGTCGTCGTCGTCGTTCCAAAGGGCTGGTGATGTTTGTCAGCACAACCCATCCAACCAGCCCGGCCAT<br>CATAAAGCTATGATCGTTTCCCGGCATCGCCCCACACCGCTGCACGGAGCAAAGCTACATAAATTTTATCCTCCGTCCAACTGGTGCA<br>CCCCCGGATCGGTACGCCGGGGATATGTGGAGAAACAGTTTGTCAACTGTCATCAAGAGCACGTGGTCcACAAGGACCACGCTTCCAC<br>AGCGGATCGGAGTTCAAACTTTTCATTTTTGGTCTAGTGTGCTGCGTGTGTGTCGCTCCATGGCACATGCGGTGTTTGTGTGTACAGG<br>TTGGGAATGCATAAAAATGTTGTTAAAATTGGACTCGTTCGTGGTGCTGTGTGTgGGAAGCTTGTTTGCCGCTGTCACTCGGCACTCG<br>GGTGACGATTACTGAGCTCTGTATGTTTGAGTTTATTTACTCGATTTGGTTTAAGCGAGAGTAGCATCGTTTCTGCTGCAGGCAAACC<br>CTTAACCAGCCCAAGAGCGTGTGTTGGAAAAAATGGGAAATGGAGGTAAATTTTAAGCACATTCCTTACAGTGCCGGGGAAATGTAAT<br>CCGGAAGTGCCCTTTTTTGGTGTTCATTTGGACATTGGACTTAATGGGCGGGAATTCCTGTTTCGAGGACGACAGTTAAAAATGCATA<br>AAAGCTGTAGTTGTAAGCCTGTGGATGTAGAATAATTCAATTTGTGAGAGAATTCATAATCTATTGCACCATTCAGGCTTGTCCTGAG<br>TTTGTTTATTTACGATGGAGTTAATCCTAATGGCGCTTAATATGATTTCAAGTATCTAAAACCTTGAGTCATCGTTTATATTAATCAT<br>TGACATTCGTACGAAGTGATTTAAAGTGCAAGTGGGGCACGCTTAACAGGCTCGTGGTGTTCAATTCTTCGTATTCAAGAGCTCTTCA<br>ACACACTCCGCGGCATGTTGGCGATATTTTGTTTTCAATATCTCTTAACACAGAGGCTCTAAAATGTGTATCGTGTTTCATGTTAGTT<br>ACGGGGCTGGTTTTTGGCTGAGAAAATCTATGCAAACATTAGGATAGTGTCACGTGTCGTATCGCTTATTATCGCACGATTTTAGATA<br>GATTTAAGTCGTCTATAAATACAACGTTCGGCTGAATTGGTGTGATCGTAGATGGCTGTGGATCATAAAACACTTCCCAACCAAACCA<br>TCAGCGGCTACTTGGGATCGTTTCTCATCTCAAGCGCGTATCAATCGCCTGATAGGGGAGGAAAATAAAACAGAACAAATCACCATcg<br>TGACGCTTACCGGCAAGATGGATGAATAGTTTTTGGCGCCATTTAAAGCGCATCAAACAGAGAGAAAAAGAGACAAAGATGGGTGGAC<br>AGAACTAAAAAAAAAATAAATCTTTTTCATTGTGGTGGAAGAATTCATCCCGGTTGCCACCAACGCAGCGTCAATGATGGTCGAATGG<br>CGTCGGTCGTTTTTTTTGGTCGATTTTATTATCACCAAAGCCGCATGACGTCCAAACATGACCGGAATTAACGAATCACCAAATCGAA<br>CGTGCAAAGGAGACGACTCCTCCCACTCCACCCCTTCCTCCGAGAAAAGCCCTGACCCGAAGCGAAGAAGCTGTCGGTGCATTTGCAC<br>CACGTTTTGCATACTGTCAGTTACAGTTCATGATTATTGCACCCTTCGCGGTTCTTCGTTCGATGCGGGAGTGTTTTTTTTTGTGTCG<br>TCACATTTATCCCACCATCGATGGCCACCCGGACCCGGGTAATGTGACCATAAAATCCCGTCACAGCGGTGGATGGAGTTGGCGGTGA<br>TATAAAATCGAACCACCCGGTTGTCGGTTGTGATCGTTGGACCGAGAATGCGTCACGTGCGTTGGGGTTTGTGTGATATTATGCACTA<br>TGGGTTTCATTAGTGTGGACGCGGGAACATTTGATTTTTTTATTTTTAATGTAATATTTAATTGCATTAATCCAGAGCTTTGTGATA<br>ACGTCACACTTTCTCACGAGCGGTCAGGGTGTGCAACTTAGTAGACTTCAAGTGTCAACGGCTTTGCTTATCCACAAGCATGAATGAA<br>CGTCGGATTGCTAAAGTACACAGTTCTGAGAGGCTCGTCATTCGATTGAAATGTGTCCAGCATCACTCATCCACTTCAGATTATCCAT<br>TGTTATCATCGCACCGAAGCATCGCAATAAAAAGGACAGACTGCACTGGCCAGTGAGTcCAGTACGGAGTGAAACGCCTTTGCCAGTA<br>CAATCTAACCGAAATAAA
**Cecropin**	ATGAACTTCTCCAAGATCTTCATCTTTGTCGTGCTGGCAGTGCTGTTGCTCTGCAGTCAGACGGAAGCGGGACGGCTGAAGAAGCTGG<br>GAAAGAAAATTGAGGGAGCCGGCAAGCGAGTGTTCAAGGCAGCAGAGAAGGCCCTACCGGTGGTGGCAGGCGTTAAGGCGCTCGGT
**linker**	GGTGGAGGTTCGGGCGGAGGCGGAACGGGTGGAGGCGGTTCCGCCCATATG
**IB2**	GAGGTGCAGCTGCAGCAGTCGGGTCCGGATCTGGTGAAGCCGGGTGCCTCGATGAAGATCTCGTGCAAGGCCTCGGGTTTCACGTTCA<br>CGGATTACAACATCCACTGGGTGAAGCAGTCGCACGGTATGTCGCTGGAGTGGATCGGTTACATCTTCCCGTACAACGGTGGTTTCGG<br>TTACAACCAGAAGTTCAAGAACAAGGCCGCCCTGACGGTGGATCACTCGTCCTCGACGGCCTACATGGAGCTGCGCTCGCTGACGTCG<br>GAGGATTCGGCCGTGTACTTCTGCACGCGCGGTGGTTACTACGGTGATTACGTGTACTTCGATGTGTGGGGTGCCGGTACGACGGTGT<br>CGGTGTCGTCGGGTGGCGGTGGATCCGACATCCAGATGACGCAGTCGCCGGCCTCGCTGTCGGTGTCGGTGGGTGAGACGGTGACGAT<br>CACGTGCCGCACGTCGGAGAACGTGTACTCGAACCTGGCCTGGTACCAGCAGAAGCAGGGTAAGTCGCCGCAGCTGCTGGTGTTCGCC<br>GCCACGAACCTGGCCGATGGTGTGCCGTCGCGCTTCTCGGGTTCGGGTTCGGGTACGCAGTTCTCGCTGAAGATCCACTCGCTGCAGT<br>CGGAGGATTTCGGTTCGTACTACTGCCAGCACTTCTGGGGTACGCCGTTCACGTTCGGTTCGGGTACGAAGCTGGATATCAAGCTCGA<br>GTCTAGAGGGCCCTTCGAAGGTAAGCCTATCCCTAACCCTCTCCTCGGTCTCGATTCTACGTGA
**AgPer3'UTR**	AGTATCGCTCAAAGTGCTCGAGCTGTTCGCCAATCGACATCGCCAAGAATGTGCTTGAAGTGTGTCACTGTGGTTAAGTAGTTGTAAG<br>AAAAATCCCCTTCTCAACTACCACAACAAGGGGTGAGTGGTTTCCTTTCATATCGATATCAGGCACTTGAGACGCAGTCGAAGACATG<br>ATCGGTCACGCTGGCATGCTTCAGGGTGGAAAGATTAGTCTAAGCAAGTGTAATAAAAGTAGGTATATAATGATGTCAGAATAGcTTG<br>CATCGCTGTTCTGTGATAATCATTACTaAAAAATAATTAAAATAGTGCGCCTAAAGTTATACTTTCATTGAGGTATGTCCAATGTCAT<br>TTAATCAAAGGTATGAGTTGAAGGGAGAATCTAATTTTATTGTATGATATACATGATTTAAATGTCGTAAAAATTGATTGACGGTGAT<br>cCAATAAAGATAAAAAGATAAACTAAAAAAGGATAGCATTATATCATTTCCTGCTTGTTCTATGGATCATGACGCCTAAATGTATGCA<br>ATGCGTTCGATTACTTATGTTATACTGTGTACAGTTTTATAGTTTTGTTTAAAAGATGTAAGGAATATAATATTTTTTGAATATTAAC<br>AAGTTGTGTTATGCTAAAATTAAAAAAAATAAATAGAAAGCGAACGAACGGGTGTAAACAATCAAGCAAATAACAATGCTCATGCTAT<br>GTCCACCATCAATACCACATTCCACTGAGGGGACAACAAAAAACAAGTGATTCGATTTCGCATCCAACCTTCACACTACCTTTAGAGA<br>CTCCATCATACTGTGTTTACTCGGGCCCACCACCAACTGACACACATTACACACGTAGTATGGGTTTAAAAGAAAAATAAGCCAAAAA<br>ATAACCGACCGaaAACTCTACCATTTCCGGTCGACCGT
**G12 3'UTR**	CGATCTTGTTTATCCGTTTGTCTCGCTCTCACTGGTGTGAAAAGATTTATTGCTTCTAGCTATTCTACGGCACGTTATGTGTCGTCAT<br>CATCGTTTGCGCAATTCCAGTTAAAATCGTATAAcAAAATTATTTcAACACGATTTTAGTGAGATTCTCCCGTTCTTTCGTAGCAATT<br>TCGTATCATTTTTGGTATTATTTTGGCGGATTTGCTTTTTTCATTCGTTTATTCCgACCTTTTCATTTGTTGCTCAAACGTTGTCCAA<br>TAGGTTTCTTATATTCTTTGACTCCTAAGTCTAAGGGGAAGAATAGTTTAGtGATGTGAATAGTAGACCACAGAATACGTTCAAGGCT<br>TATCGAAAGTGaAGGgTCAAAGCGAATTTGCGAAgAAGTGTTTAAGAGCGTATTTCTCAACGTACAATAGTTCATtTCAGCTTcgAAT<br>ATGATTTcAATGAGTCTTTATTCAGTATGTGCTTCCATTGcCATTCCATTGGTGTTCGTTCGAT
**m4B7-HA tag**	CTAAGCGTAATCTGGAACATCGTATGGGTATGCGGCCGCCCGTTTGATCTCCAGTTTGGTGCCGCTGCCAAAGGTCAGCGGATACGCA<br>TTGCGCTGCAGACAGTAGTAATCCGCAAAGTCCTCGCTTTCCAGCGACGAAATCGTCAGCGAATAATCCGAGCCGCTGCCGCTGCCGC

	TAAAGCGCGACGGCACGCCCGAATTCAGATTCGAGGTGCTGTAGATCAGCAGCTTAATCGTGCCGCCCGGCTTCTGTTGAAACCAATC<br>CAGGTTGCCCCGAATGTCCTGGCTGGCCCGACACGACAGGCTGACGCGGTCGCCCAGGCTCGCGAACATGCTCGAGGGCGACTGCGTC<br>ATCTCGATGTCCGAACCACCACCGCCCGAGCTCACCGTCACCGTGGTGCCCTGACCCCAGTAGTCCAGGCCGTAGTACAGGTTGCCCC<br>GCGCGCAGTAGTACATCGCGGTATCTTCCGAGCGCAGGCTCGACATCTCCAGGTACAGCGTGTTTTTCGCGTTCTCGCGCGAAATGGT<br>GAAGCGGCCCGTCACCGTGTCGGCATAGTAGATCGAGTAGGCCAGGTTGTTGATGAACGCGACCCACTCCGGACCTTTACCGGGGGCC<br>TGGCGGAACCACGCCATGCCGTAATCGCTGAAGGTGAAGCCCGACGCCGCGCACGACAGCTTCCGCGAGCCGCCGGGCTGCACCAGGC<br>CACCACCGCTCTCCACCAGCTTCACcat
**G12-5'UTR**	GATGCTAGATTTGATTGGGTTGCTTTTGCACTGGTTTCGATGCTGTGGTACTGCTGTACTGAGCTGTAGTAGGCTTGGGAATGATTTC<br>TCTACCAACCAGCCAGTAGCTTTTATACGTCCGCTGCTGTCAGTATCTTGAGCGGTCAACAGTCGATTAACCAGCTGTCGAAGGAGTG<br>GAGTGCTGTTATCTGTGCCTcTCGAGTGTACATCTGTTGGCAacTACTGGACTTTCAGCCgGTCTGTCCCACACTCACTCTCAAGAAG<br>TCCACCGtCCACTACCCATCACTGGTCAAGTGGTCCATACTTATTATAATAACATAATCCGATTAGCGATAATGATGATTTAGCATGG<br>AATTTGTGTaCTTAATTGATTACTGATCACGCCCTTTTgGTTTGGATGGGAACTTcTTCCCTTGGAACTCTCtACTTACcGTTCAATC<br>CAGTACTTCTTCTGCGAACAGATGTTTCTAAGGCAACCGATAAGGCCAACCTGGACCTTGGACACAGTTCGTTGCAATGGAATGATTT<br>tCACATGATTTAGTTTCTGGATGGAGATCTTCAGTCTAGTGGCTGGCTACTTGGTCGcAGCTAAATTTCTACGCTGcGTTTTTCCTCT<br>AAAGTACtGctGGGAATGGTTGgTTCACCAGTAGGaGAGATCAGTTGCCGaATTgGAGCAGtAGGTTTGGTATCGGtTTCTTGTTTCG<br>GGACAGGTTCACtGATTCGTTCTTTCACTGAGATTGGTCTATGGTACAGTTTTCCATGAATTGGAATAAAGTAGGgATGAGTTCTAGG<br>ATCAGTTTGcCATGGGAAAAATCCAGGACCAGTATAGGTTATGGATCAATTCCAGGATCgATATGGATAtGGAATcAGTTTGtGAAC<br>CGGTGAGGATTCtGTAgaAAaTTCCTGAATCGGTATGGtTGGCTTAGGGATCATTTCGTAGACCAATATGAGTTTACTATCAGTaTCG<br>GGAATATTATCAAATTTGaACAAGTTCCTAGGATGGGTACAGTATCTGTTCCAGGACTGGTATGGGTACTTCTGGGACTTCCGGGATT<br>TCAAAGAATGGTATCGTTTCATGGTCGGTTCCAGGACTGACCTGGGCTCAGTTTCTTAATCAaTTTCAGGGCTGGTACGTGAGTAGGA<br>TAGAGTTCCAAGACTAGAATGGATTGAGTTCTAGGACCTAATTAGTTGCTGGATCGCTAAACTAGTTTAAGTTTAGGATTATTACCaG<br>cTCTTTTAAAGATTCAGGGCCGGTATTGTGGTACAGTTGTTAACTCGTACAACAGCATACCCGGCATGGATTCAAGCCCCGAATGGAC<br>CGTGTCATAGGACTGACTATCCTGCTATGGGTAAcCAATAAGGCACTGAAAGCCAAGCCTACCAGgCAGGTATTGATCGACAACCGTT<br>GTTGTgCCAAGTAGAaAAAAAAGATTCAATATCAGTTCTAGGACCGCCATcgaacatcgattctaggatcGAAATCGAACAAATCCTA<br>GCAACAGCATTGGATCAAAATAAGCTCCACGATCAGTATGGATTCAGGATCTATGCCTGGACTTGTATGGGATCAGTATCAGAGTTTC<br>AGGATCAG
**3xp3**	GAGCTCGCCCGGGGATCTAATTCAATTAGAGACTAATTCAATTAGAGCTAATTCAATTAGGATCCAAGCTTATCGATTTCGAACCCTC<br>GACCGCCGGAGTATAAATAGAGGCGCTTCGTCTACGGAGCGACAATTCAATTCAAACAAGCAAAGTGAACACGTCGCTAAGCGAAAGC<br>TAAGCAAATAAACAAGCGCAGCTGAACAAGCTAAACAATCGGGGTACCGCTAGAGTCGACGGTACCGCGGGCCCGGGATCCACCGGTC<br>GCCACC
**DsRed1**	ATGGTGCGCTCCTCCAAGAACGTCATCAAGGAGTTCATGCGCTTCAAGGTGCGCATGGAGGGCACCGTGAACGGCCACGAGTTCGAGA<br>TCGAGGGCGAGGGCGAGGGCCGCCCCTACGAGGGCCACAACACCGTGAAGCTGAAGGTGACCAAGGGCGGCCCCCTGCCCTTCGCCTG<br>GGACATCCTGTCCCCCCAGTTCCAGTACGGCTCCAAGGTGTACGTGAAGCACCCCGCCGACATCCCCGACTACAAGAAGCTGTCCTTC<br>CCCGAGGGCTTCAAGTGGGAGCGCGTGATGAACTTCGAGGACGGCGGCGTGGTGACCGTGACCCAGGACTCCTCCCTGCAGGACGGCT<br>GCTTCATCTACAAGGTGAAGTTCATCGGCGTGAACTTCCCCTCCGACGGCCCCGTAATGCAGAAGAAGACCATGGGCTGGGAGGCCTC<br>CACCGAGCGCCTGTACCCCCGCGACGGCGTGCTGAAGGGCGAGATCCACAAGGCCCTGAAGCTGAAGGACGGCGGCCACTACCTGGTG<br>GAGTTCAAGTCCATCTACATGGCCAAGAAGCCCGTGCAGCTGCCCGGCTACTACTACGTGGACTCCAAGCTGGACATCACCTCCCACA<br>ACGAGGACTACACCATCGTGGAGCAGTACGAGCGCACCGAGGGCCGCCACCACCTGTTCCTGTAG
**HA2 SWAP**	CTCTAGATCATAATCAGCCATACCACATTTGTAGAGGTTTTACTTGCTTTAAAAAACCTCCCACACCTCCCCCTGAACCTGAAACATA<br>AAATGAATGCAATTGTTGTTGTTAACTTGTTTATTGCAGCTTATAATGGTTACAAATAAAGCAATAGCATCACAAATTTCACAAATAA<br>AGCATTTTTTTCACTGCATTCTAGTTGTGGTTTGTCCAAACTCATCAATGTATCTTAAAGCTTATCGATACGCGTACGGCGCGCCggc<br>cgtgtggtttaacccaccaaactggcagtttcttggctttgttcgatgcaactggatttttagtacaggaaattggtggcgttttcgg<br>tatcttggttatttgggtagtgtatagtggtgttttcgaaattcatttttgttcattaacagttgttcaacctatagttatttattac<br>aataatattctactaacgattaaccgattgattcaaagtgaataaattataaaactagtgattttttttTaaatttttgtatgatttt<br>gtcatttcttggatcattatccgtgcaaatcgattaaccggcaacagtccggtcccgagctgcccgaataatcgacgttctactgtat<br>tcctaccgacttttgatatgcctactgactcaccgatgagccccctaaaactaccgatttttatttatcctaccgaaaatcacaga<br>ttttttcgtaatactgaccaaaaagtcatgtaatcatttcccaaatcacttaatgtattaaactccatatggaaatcactagcaacca<br>gaacaagaagttcaacagagacaacccccgtgtacttcatgagatgcgatgatatcggacgcgctggtaaaatttatatggtatttg<br>aaagatactgtaagacgtgcgatttttaatacgatggaatcaattcaacagtcaattatgaattaattaagaatcaatttgtagaaa<br>aaatctctaatgtatgttttgttatcgtaacagttaccaattcatccaaataatcgacatagcgacacagcacattcgatttagcaat<br>gtcctaagcggccgcgtggagccccaaaaaaagggagtccgcaacgcttagaagcaaatgtgcatctcaatcattcgttggg
**pTP43**	
**HA1 SWAP (same as Ag-card HM1)**	CTGGAACAGCCGCTCGTCGTCCCAGTGCGGGTTTGCGCGGGCCAGCCCGCGGGCAAGGCTGTTGTGGTGCCGGGCCCAGATCAGATGC<br>ATCGAGGTGAGATGCAGATTCTCGTTCGCCCGCGCATCGCCCGACTCGAAGCAGTACTTGCCGGCCGCATTCATCTCCTGCTCGTTGC<br>ACCCGTCGAGCGGATCGGTCGACACGGGCAGCAGATCGCGACCGTCCGGCGTGCGCAGCATACGCAGCTGGCCACCGGCGCCCGTGCG<br>CAGCGCGCCCATACGCTCGTCGTCCGAGCCGTACACGACCGAGCCGTCGATGAAGGCGGTCGCCTGGTTCAGCTGCTGCCGCGGACCG<br>AAGTGACCGGTCGGGGCCGGCACCGAGCGCACAAAGTTCATGCAGGTCACGTTGTACTGGGTGTAGTACGGGTCGCCCGGACCGAGCG<br>GCACGGGAAAGCACTCGGGATGTTGCGGCTGCCCCGGATCGCAGCACTCGATCGGCTTCCCGTCCACGCCCTGGTTCAGCGCGGTGGA<br>GGTTATGTCGTGATCGAGAAACTGGCCCCACACCGCCAGCATCACGCTAAAGTTCGGATCGTTGTGGTACGATGGCGATGGATTTCG<br>AGCGACACCTGCCGGGCGCTGGGCAGTTCGGCCCCATCGACGGAGGCACGCGGTGCCGATATGCCATCGCCATAGTCCGGGTTGAGCT<br>GGCGCCGGAACGGTATCATCGCGACGCCGTACTCGTACGGTCGCTCCTTACTGTTGCACGTCCCGTTGGCCGTGCGATAGCGCGCGTT<br>AAAGTCACAGCGCGGTTGACGGTGCGCACGCGGCAAATCCATCGGTGGGCCGCGCCCGATCGAGCCCCGACGACGGATGTCCAACCGG<br>CGCACCAGTGCCTGCGTGGCACGATCCTCCACGAAGCCGACCTTTGCCGCCAGCCGGGCCGCTTTCGTTGCACCGACGGACTTTTGGT<br>GGCGGAAGGAGGGCGAGTTAAGCGGGGGCGACGAGAGCGTCTCCTCCAGCAGCTCCTTATCGCCCAGCGCCTTCTCGCCATCCGCTAT<br>GGCGGCCGCCT
**G12 3'UTR**	CGATCTTGTTTATCCGTTTGTCTCGCTCTCACTGGTGTGAAAAGATTTATTGCTTCTAGCTATTCTACGGCACGTTATGTGTCGTCAT<br>CATCGTTTGCGCAATTCCAGTTAAAATCGTATAAcAAAATTATTTcAACACGATTTTAGTGAGATTCTCCCGTTCTTTCGTAGCAATT<br>TCGTATCATTTTTGGTATTATTTTGGCGGATTTGCTTTTTTCATTCGTTTATTCCgACCTTTTCATTTGTTGCTCAAACGTTGTCCAA<br>TAGGTTTCTTATATTCTTTGACTCCTAAGTCTAAGGGGAAGAATAGTTTAGtGATGTGAATAGTAGACCACAGAATACGTTCAAGGCT<br>TATCGAAAGTGaAGGgTCAAAGCGAATTTGCGAAgAAGTGTTTAAGAGCGTATTTCTCAACGTACAATAGTTCATtTCAGCTTcgAAT<br>ATGATTTcAATGAGTCTTTATTCAGTATGTGCTTCCATTGcCATTCCATTGGTGTTCGTTCGAT
**Scorpine-V5-6Hxis**	TTAGTGGTGGTGGTGGTGGTGCTCGAGcgtagaatcgagaccgaggagagggttagggataggcttaccataagacagggggggtgccg<br>catttacacttggtgccgtgacagtagcctttttcgccgctcgtctgacagtgtttctcgcaattgcccagcatatccatgttcgcca<br>tgcactgaaattcgttcttggccattttatgcacaatcgctttcgccatgcgacccaGgaccgtgttgcccatccgttcgtcaatctt<br>tttctgaatcttctcttcgttaatccagcC

**SHIVA-Myc-P2A**	GGGGCCGGGGTTCTCCTCCACGTCGCCGGCCTGCTTCAGCAGGGAGAAGTTGGTGGCCAGATCCTCTTCTGAGATGAGTTTTTGTTCa<br>cccaccgcacgcgcatcgccaaccagcgcaatcgccggacccgcacgcagaaTaccctgcttaatctgtttgccaacgcggtcgatac<br>ggcggaacagacgccaacgcggcaT
**(EPIP)4-HA-P2A**	GGGGCCGGGGTTCTCCTCCACGTCGCCGGCCTGCTTCAGCAGGGAGAAGTTGGTGGCAGCGTAATCTGGAACATCGTATGGGTAgccc<br>ggcgagcccttcaccagcgatttgtcgcccggcgagcccttgaccaggcttttatcggggccgcccggacctttgaccagcgatttgt<br>CcgggccgcccggacccttaaccagggatttatC
**(TP10)2-FLAG-P2A**	GGGGCCGGGGTTCTCCTCCACGTCGCCGGCCTGCTTCAGCAGGGAGAAGTTGGTGGCCTTGTCGTCATCGTCTTTGTAGTCcaggatc<br>ttcttggccagggcggccagggccttcaggttgatcttgcccagcaggtagccggcgccgcccaggatcttcttggccagggcggcca<br>gggccttcaggttgatcttgcccagcaggtagccggc
**Melittin-6xHis-P2A**	GGGGCCGGGGTTCTCCTCCACGTCGCCGGCCTGCTTCAGCAGGGAGAAGTTGGTGGCatggtgatggtgatgatgctgctggcgcttg<br>cgcttgatccacgagatcagggccggcaggcccgtcgtcagcaccttcagcacggcgccgatgcc
**AgCp-SP (signal peptide)**	TGGATCCGCCTCGGCCGCTTCGACACTGAGCGCCACCGCCAGGATGGCCAT
**G12-5'UTR**	GATGCTAGATTTGATTGGGTTGCTTTTGCACTGGTTTCGATGCTGTGGTACTGCTGTACTGAGCTGTAGTAGGCTTGGGAATGATTTC<br>TCTACCAACCAGCCAGTAGCTTTTATACGTCCGCTGCTGTCAGTATCTTGAGCGGTCAACAGTCGATTAACCAGCTGTCGAAGGAGTG<br>GAGTGCTGTTATCTGTGCCTcTCGAGTGTACATCTGTTGGCAacTACTGGACTTTCAGCCgGTCTGTCCCACACTCACTCTCAAGAAG<br>TCCACCGtCCACTACCCATCACTGGTCAAGTGGTCCATACTTATTATAATAACATAATCCGATTAGCGATAATGATGATTTAGCATGG<br>AATTTGTGTaCTTAATTGATTACTGATCACGCCCTTTTgGTTTGGATGGGAACTTcTTCCCTTGGAACTCTCtACTTACcGTTCAATC<br>CAGTACTTCTTCTGCGAACAGATGTTTCTAAGGCAACCGATAAGGCCAACCTGGACCTTGGACACAGTTCGTTGCAATGGAATGATTT<br>tCACATGATTTAGTTTCTGGATGGAGATCTTCAGTCTAGTGGCTGGCTACTTGGTCGcAGCTAAATTTCTACGCTGcGTTTTTCCTCT<br>AAAGTACtGctGGGAATGGTTGgTTCACCAGTAGGaGAGATCAGTTGCCGaATTgGAGCAGtAGGTTTGGTATCGGtTTCTTGTTTCG<br>GGACAGGTTCACtGATTCGTTCTTTCACTGAGATTGGTCTATGGTACAGTTTTCCATGAATTGGAATAAAGTAGGgATGAGTTCTAGG<br>ATCAGTTTGcCATGGGGAAAAATCCAGGACCAGTATAGGTTATGGATCAATTCCAGGATCgATATGGATAtGGAATcAGTTTGtGAAC<br>CGGTGAGGATTCtGTAgaAAaTTCCTGAATCGGTATGGtTGGCTTAGGGATCATTTCGTAGACCAATATGAGTTTACTATCAGTaTCG<br>GGAATATTATCAAATTTGaACAAGTTCCTAGGATGGGTACAGTATCTGTTCCAGGACTGGTATGGGTACTTCTGGGACTTCCGGGATT<br>TCAAAGAATGGTATCGTTTCATGGTCGGTTCCAGGACTGACCTGGGCTCAGTTTCTTAATCAaTTTCAGGGCTGGTACGTGAGTAGGA<br>TAGAGTTCCAAGACTAGAATGGATTGAGTTCTAGGACCTAATTAGTTGCTGGATCGCTAAACTAGTTTAAGTTTAGGATTATTACCaG<br>cTCTTTTAAAGATTCAGGGCCGGTATTGTGGTACAGTTGTTAACTCGTACAACAGCATACCCGGCATGGATTCAAGCCCCGAATGGAC<br>CGTGTCATAGGACTGACTATCCTGCTATGGGTAAcCAATAAGGCACTGAAAGCCAAGCCTACCAGgCAGGTATTGATCGACAACCGTT<br>GTTGTgCCAAGTAGAaAAAAAAGATTCAATATCAGTTCTAGGACCGCCATcgaacatcgattctaggatcGAAATCGAACAAATCCTA<br>GCAACAGCATTGGATCAAAATAAGCTCCACGATCAGTATGGATTCAGGATCTATGCCTGGACTTGTATGGGATCAGTATCAGAGTTTC<br>AGGATCAG
**hr5 enhancer**	cgcgtaaaacacaatcaagtatgagtcataatctgatgtcatgttttgCacacggctcataaccgaactggctttacgagtagaattc<br>tacttgtaaCgcacgatcagtggatgatgtcatttgtttttcaaatcgagatgatgtcatgttttgcacacggctcataaactcgctt<br>tacgagtagaattctacgtgtaacgcacgatcgattgatgagtcatttgttttgcaatatgatatcatacaatatgactcatttgttt<br>ttcaaaaccgaacttgatttacgggtagaattctacttgtaaagcacaatcaaaaagatgatgtcatttgtttttcaaaactgaactc<br>gctttacgagtagaattctacgtgtaaaacacaatcaagaaatgatgtcatttgttataaaaataaaagctgatgtcatgttttgcac<br>atggctcataactaaactcgctttacgggtagaattctacgcg
**IE1**	atttgcagttcgggacataaatgtttaaatatatcgatgtctttgtgatgcgcgcgacatttttgtaggttattgataaaatgaacgg<br>atacgttgcccgacattatcattaaatccttggcgtagaatttgtcgggtccattgtccgtgtgcgctagcatgcccgtaacggacct<br>cgtacttttggcttcaaaggttttgcgcacagacaaaatgtgccacacttgcagctctgcatgtgtgcgcgttaccacaaatcccaac<br>ggcgcagtgtacttgttgtatgcaaataaatctcgataaaggcgcggcgcgcgaatgcagctgatcacgtacgctcctcgtgttccgt<br>tcaaggacggtgttaCcgacctcagattaatgtttatcggccgactgttttcgtatccgctcaccaaacgcgtttttgcattaacatt<br>gtatgtcggcggatgttctatatctaatttgaataaataaacgataaccgcgttggttttagagggcataataaaagaaatattgtta<br>tcgtgttcgccattagggcagtataaattgacgttcatgttggatattgtttcagttgcaagttgacactggcggcgacaagAcaatt<br>ctaattggggtaagttttcccgttcttttctgggttcttccctttttgctcatccttgctgcactaccttcaggtgcaagttgagattc<br>aggccaccatgggagatcccaccccacccaagaagaagcgcaaaccggtcgccacc
**mCherry**	ATGGTGAGCAAGGGCGAGGAGGATAACATGGCCATCATCAAGGAGTTCATGCGCTTCAAGGTGCACATGGAGGGCTCCGTGAACGGCC<br>ACGAGTTCGAGATCGAGGGCGAGGGCGAGGGCCGCCCCTACGAGGGCACCCAGACCGCCAAGCTGAAGGTGACCAAGGGTGGCCCCCT<br>GCCCTTCGCCTGGGACATCCTGTCCCCTCAGTTCATGTACGGCTCCAAGGCCTACGTGAAGCACCCCGCCGACATCCCCGACTACTTG<br>AAGCTGTCCTTCCCCGAGGGCTTCAAGTGGGAGCGCGTGATGAACTTCGAGGACGGCGGCGTGGTGACCGTGACCCAGGACTCCTCCC<br>TGCAGGACGGCGAGTTCATCTACAAGGTGAAGCTGCGCGGCACCAACTTCCCCTCCGACGGCCCCGTAATGCAGAAGAAGACCATGGG<br>CTGGGAGGCCTCCTCCGAGCGGATGTACCCCGAGGACGGCGCCCTGAAGGGCGAGATCAAGCAGAGGCTGAAGCTGAAGGACGGCGGC<br>CACTACGACGCTGAGGTCAAGACCACCTACAAGGCCAAGAAGCCCGTGCAGCTGCCCGGCGCCTACAACGTCAACATCAAGTTGGACA<br>TCACCTCCCACAACGAGGACTACACCATCGTGGAACAGTACGAACGCGCCGAGGGCCGCCACTCCACCGGCGGCATGGACGAGCTGTA<br>CAAGTAA
**HA2 SWAP**	CTCTAGATCATAATCAGCCATACCACATTTGTAGAGGTTTTACTTGCTTTAAAAAACCTCCCACACCTCCCCCTGAACCTGAAACATA<br>AAATGAATGCAATTGTTGTTGTTAACTTGTTTATTGCAGCTTATAATGGTTACAAATAAAGCAATAGCATCACAAATTTCACAAATAA<br>AGCATTTTTTTCACTGCATTCTAGTTGTGGTTTGTCCAAACTCATCAATGTATCTTAAAGCTTATCGATACGCGTACGGCGCGCCggc<br>cgtgtggtttaacccaccaaactggcagtttcttggctttgttcgatgcaactggatttttagtacaggaaattggtggcgttttcgg<br>tatcttggttatttgggtagtgtatagtggtgttttcgaaattcatttttgttcattaacagttgttcaacctatagttatttattac<br>aataatattctactaacgattaaccgattgattcaaagtgaataaattataaaactagtgattttttttTaaatttttgtatgatttt<br>gtcatttcttggatcattatccgtgcaaatcgattaaccggcaacagtccggtcccgagctgcccgaataatcgacgttctactgtat<br>tcctaccgacttttgatatgcctactgactcaccgatgagcccccctaaaactaccgatttttatttatcctaccgaaaatcacaga<br>ttttttcgtaatactgaccaaaaagtcatgtaatcatttcccaaatcacttaatgtattaaactccatatggaaatcactagcaacca<br>gaacaagaagttcaacagagacaaccccccgtgtacttcatgagatgcgatgatatcggacgcgctggtaaaattttatatggtatttg<br>aaagatactgtaagacgtgcgatttttttaatacgatggaatcaattcaacagtcaattatgaattaattaagaatcaatttgtagaaa<br>aaatctctaatgtatgttttgttatcgtaacagttaccaattcatccaaataatcgacatagcgacacagcacattcgatttagcaat<br>gtcctaagcggccgcgtggagccccaaaaaaagggagtccgcaacgcttagaagcaaatgtgcatctcaatcattcgttggg

REFERENCES

Carballar-Lejarazú, R., Dong, Y., Pham, T. B., Tushar, T., Corder, R. M., Mondal, A., Sánchez C, H. M., Lee, H. F., Marshall, J. M., Dimopoulos, G., & James, A. A. (2023). Dual effector population modification gene-drive strains of the African malaria mosquitoes, *Anopheles gambiae* and *Anopheles coluzzii*. *Proc. Natl. Acad. Sci. USA*. 120(29), e2221118120. https://doi.org/10.1073/pnas.2221118120

Dong, Y., Simões, M. L., & Dimopoulos, G. (2020). Versatile transgenic multistage effector-gene combinations for *Plasmodium falciparum* suppression in *Anopheles*. *Science advances*, *6*(20), eaay5898. https://doi.org/10.1126/sciadv.aay5898
